# Artificial Intelligence-Enhanced Wearable Blood Pressure Monitoring in Resource-Limited Settings: A Co-Design of Sensors, Model, and Deployment

**DOI:** 10.1007/s40820-025-02003-9

**Published:** 2026-01-05

**Authors:** Yiming Zhang, Shirong Qiu, Kai Du, Shun Wu, Ting Xiang, Kenghao Zheng, Zijun Liu, Hanjie Chen, Nan Ji, Fa Wang, Weijia Wu, Yuan-Ting Zhang

**Affiliations:** 1 Department of Electronic Engineering, The Chinese University of Hong Kong, Sha Tin, 999077 Hong Kong SAR People’s Republic of China; 2 College of Electronic and Information Engineering, Southwest University, Chongqing, 400715 People’s Republic of China; 3 Department of Biomedical Engineering, City University of Hong Kong and Hong Kong Centre for Cerebro-Cardiovascular Health Engineering (COCHE), Sha Tin, 999077 Hong Kong SAR People’s Republic of China; 4 United Imaging Microelectronics Technology, Shanghai, 201815 People’s Republic of China; 5 Department of Electrical and Computer Engineering, National University of Singapore, Singapore, Singapore

**Keywords:** Wearable blood pressure, Resource-limited, EdgeAI, Cardiovascular health

## Abstract

Accurate blood pressure (BP) monitoring is essential for preventing and managing cardiovascular disease. Advancements in materials science, medicine, flexible electronic, and artificial intelligence (AI) have enabled cuffless, unobtrusive BP monitoring systems, offering an alternative to traditional sphygmomanometers. However, extending these advances to real-world cardiovascular care particularly in resource-limited settings remains challenging due to constraints in computational resources, power efficiency, and deployment scalability. This review presents a comprehensive synthesis of AI-enhanced wearable BP monitoring, emphasizing its potential for personalized, scalable, and accessible healthcare. We systematically analyze the end-to-end system architecture, from mechano-electric sensing principles and AI-based estimation models to edge-aware deployment strategies tailored for low-resource environments. We further discuss clinical validation metrics and implementation barriers and prospective strategies. To bridge lab-to-field translation, we propose an innovative "sensor-model-deployment-assessment" co-design framework. This roadmap highlights how AI-enhanced BP technologies can support proactive hypertension control and promote cardiovascular health equity on a global scale.

**Supplementary Information:**

The online version contains supplementary material available at 10.1007/s40820-025-02003-9.

## Highlights

- Integrative Co-Design Framework: We synthesize current advances in sensing, models, accuracy/reliability assessment, and hardware into a sensor–model–deployment–assessment framework that organizes evidence and design trade-offs for cuffless blood pressure monitoring. The framework seeks to balance precision and efficiency by jointly considering low-power edge AI, streamlined sensor architectures, and adaptive computational models, providing a structured basis for reproducible and clinically meaningful wearable solutions.
- Pathways to Clinical Translation: We critically assess barriers to real-world deployment, offering actionable strategies to bridge the translational gap between laboratory innovations and scalable implementation in low-resource regions with minimal healthcare infrastructure.
- Interdisciplinary Synthesis: By integrating cutting-edge advances in materials science, digital health, and embedded AI, we provide evidence-based recommendations to empower biomedical researchers, engineers, and data scientists in advancing equitable diagnostic solutions.

**Supplementary Information:**

The online version contains supplementary material available at 10.1007/s40820-025-02003-9.

## Graphical abstract

**Figure:**
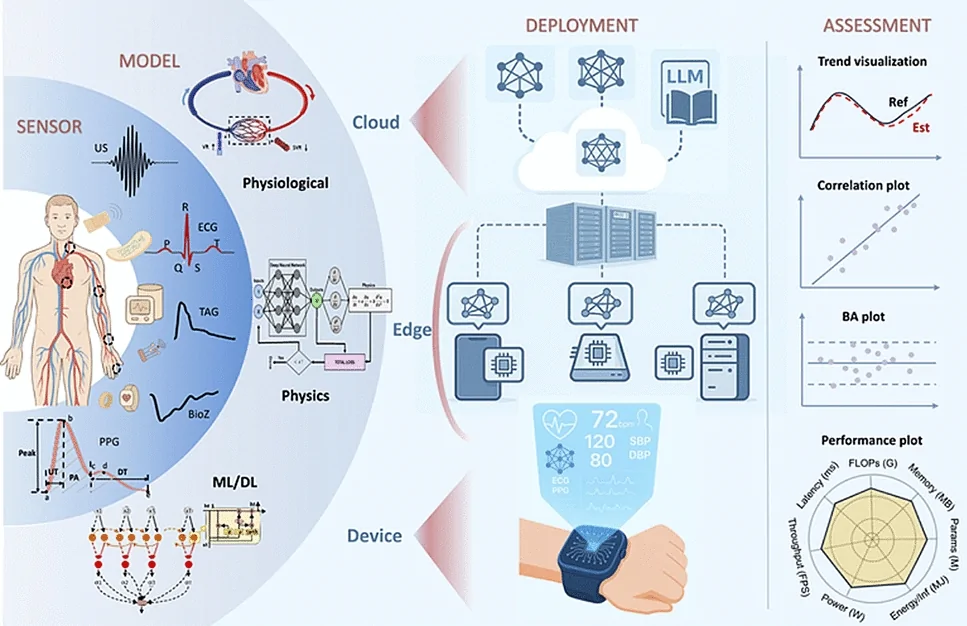

## Introduction

Hypertension is a major risk factor for cardiovascular diseases (CVDs), contributing significantly to global morbidity and mortality [1]. Accurate and continuous blood pressure (BP) monitoring is thus essential for early diagnosis, preventive care, and personalized intervention [2], particularly in resource-limited settings where access to episodic clinical measurement is limited. Traditional cuff-based BP measurement [3], despite its clinical acceptance, remains inherently episodic, cumbersome, and ill-suited for unobtrusive long-term monitoring [4]. Cuffless BP estimation represents a paradigm shift in non-invasive monitoring by eliminating the need for traditional cuffs, supporting cost-effective, continuous BP monitoring during daily life and holds potentials for personalized, proactive hypertension management [5].

Recent advances in sensing technologies have further empowered this field, enabling the acquisition of high-quality physiological data through increasingly miniaturized and affordable wearable devices [6–9]. Concurrently, artificial intelligence (AI) has emerged as a transformative tool for analyzing these complex signals, significantly enhancing the accuracy and robustness of cuffless BP estimation [8]. These trends have created new opportunities for deploying AI-driven BP monitoring beyond traditional healthcare settings. In particular, resource-limited settings—including low- and middle-income countries (LMICs), remote communities, and underserved populations in high-income countries—represent environments where the potential impact of wearable BP monitoring is especially high [6, 10, 11]. These settings are often characterized by limited healthcare infrastructure, insufficient access to trained personnel, and high unmet needs for hypertension screening and management. Yet, deploying state-of-the-art AI-based BP estimation methods in such settings requires a fundamental rethinking of system design. Existing approaches [12, 13] typically assume ample computational resources and high-quality signals, whereas real-world deployments in resource-constrained settings must navigate variable signal quality, limited model capacity, strict energy budgets, intermittent connectivity, and fragmented data ecosystems [14]. Scalable and reliable BP monitoring in such environments demands a delicate balance between model accuracy, algorithmic complexity, and hardware efficiency [15–17]. It requires coordinated advances in sensor technology, learning frameworks, and edge-aware system deployment tailored to the realities of diverse populations and care infrastructures.

**Fig. 1:**
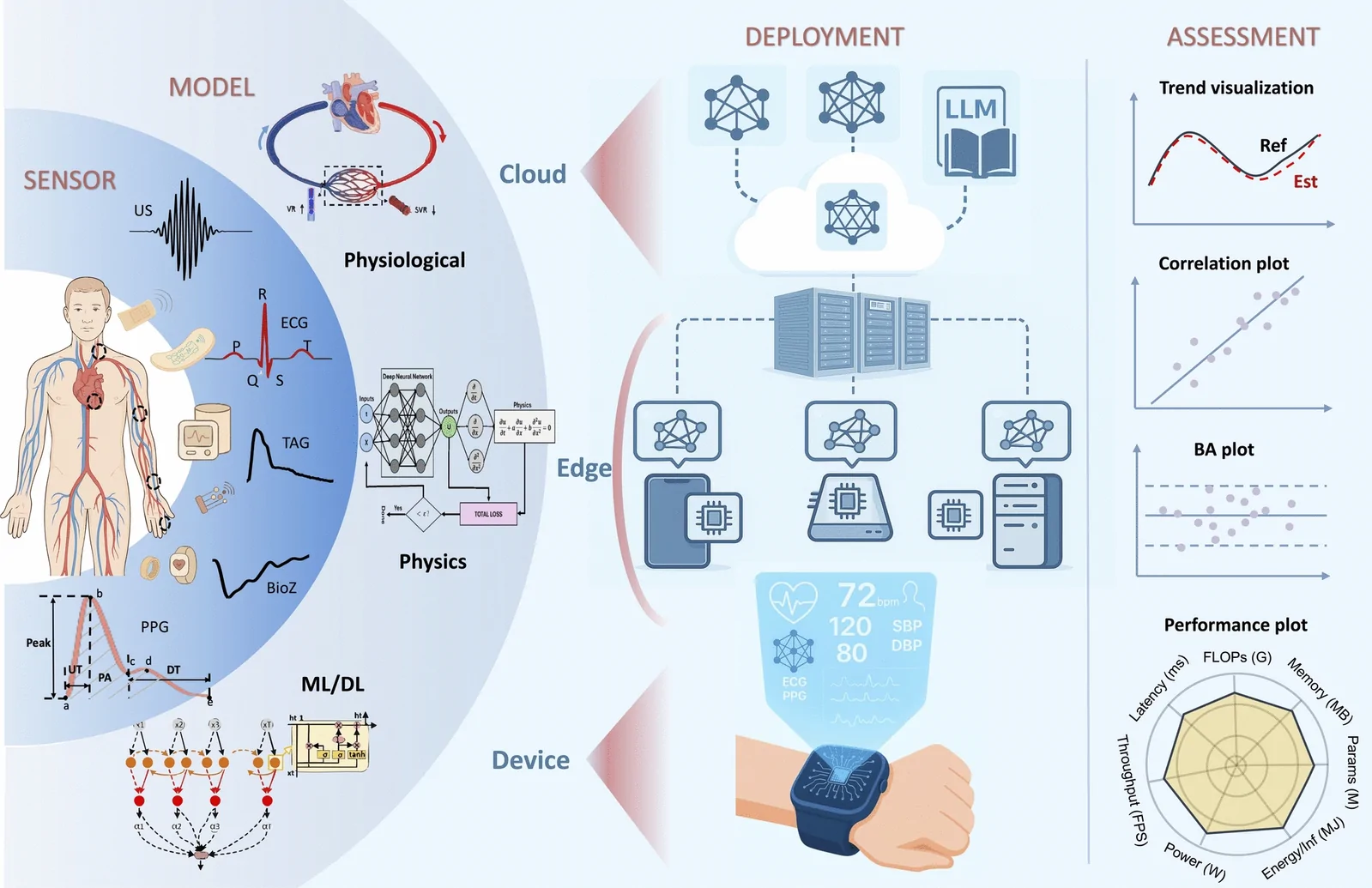
The sensor–model–deployment–assessment framework for AI-based cuffless BP estimation in real-world resource constraints

In this review, we provide an integrated perspective on the co-design of sensing, modeling, deployment and assessment, which is critical in real-world deployments but often has been neglected in prior reviews [7, 9, 18]. To be specific, we will systematically analyze the infrastructure, model, and deployment challenges of AI-based blood pressure estimation in resource-limited settings and summarize promising solutions and emerging directions for scalable and accessible healthcare. Figure 1 shows a system view that spans from hardware-proximal sensing, model design to execution across device–edge–cloud framework and comprehensive assessment. First, advanced wearable sensing technologies such as optical [19], electrical [20], mechanical [21], acoustic [22], and electromagnetic [23] enable various physiological signals acquisition. Second, the captured signals are further processed using physiological-based models, physics-based models, and data-driven machine learning models. These models are employed to estimate BP in three clinical scenarios, high-demand: beat-to-beat or BP waveform [24] for stress tests and acute monitoring; moderate-demand: intermittent BP for ambulatory follow-up and therapy titration; and low-demand: snapshot BP for spot checks. During the hardware deployment phase, challenges related to model optimization, compilation, scheduling, and adaptability across heterogeneous hardware platforms (microcontrollers, mobile devices, edge servers, and cloud platforms) will be discussed. Finally, we provide a comprehensive, system-level evaluation from both the model and device perspectives.

## Wearable Sensing Technology for BP Measurement

We start with a concise overview of wearable sensing technologies for BP measurement, including photoplethysmography, electrical, acoustic, mechano-electric, and radar methods, focusing on their material innovation, sensor design, principles, performance, and limitations.

**Fig. 2:**
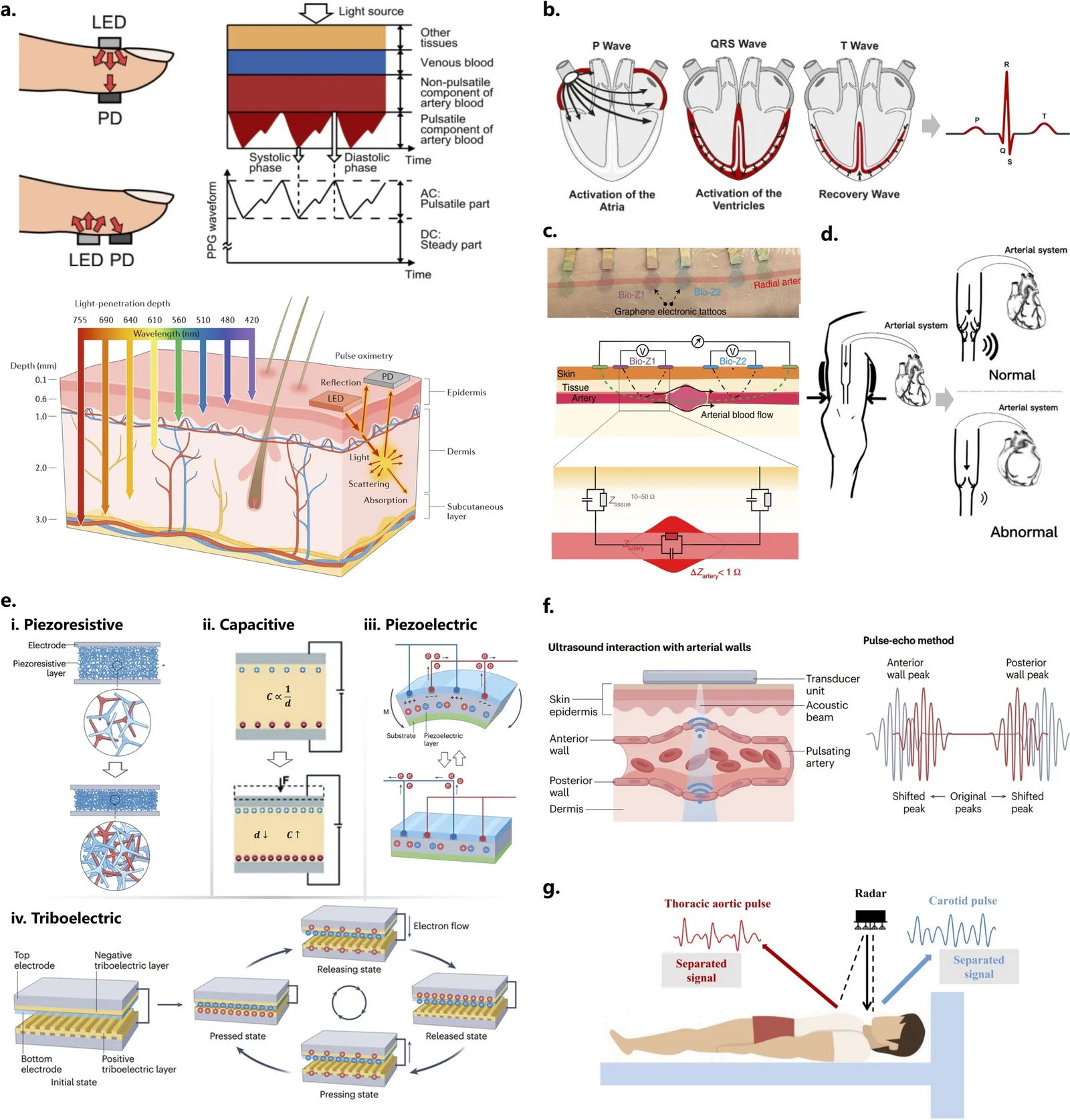
Working principle of wearable BP sensing. **a** Optical sensing (PPG). Adapted from [40], Electronics, 2014, published by MDPI, under the terms of the Creative Commons CC-BY license. Adapted with permission from [41], Copyright Springer Nature, 2019. **b** ECG sensing. Adapted from https://www.capitalheart.sg/what-does-an-abnormal-ecg-mean/. **c** Bioimpedance sensing. Adapted with permission from [32], Copyright Springer Nature, 2022. **d** Korotkoff sounds. Adapted from [22], Frontiers in Cardiovascular Medicine, 2022, under the terms of the Creative Commons CC-BY license. **e** Mechano-electric sensing, including piezoresistive, capacitive, piezoelectric, and triboelectric sensing. i), iii), and iv) Adapted with permission from [7], Copyright Springer Nature, 2025. ii) Adapted from [42], with permission from the Royal Society of Chemistry. **f** Ultrasound sensing. Adapted with permission from [7], Copyright Springer Nature, 2025. **g** Radar sensing. Adapted with permission from [23], Copyright Elsevier, 2023

Photoplethysmography (PPG) is a cost-effective and non-invasive optical technique that measures changes in peripheral blood volume to monitor cardiovascular parameters (Fig. 2a). The fundamental operation involves light-emitting diodes (LEDs) illuminating tissue and photodetectors capturing backscattered or transmitted light, the intensity of which is modulated by cardiac cycle-induced blood flow variations. It is noteworthy that over 95% of the total detected light intensity originates from static tissue compartments (e.g., dermis, subcutaneous fat, muscle), with only a small variable component attributable to pulsatile blood volume changes, underscoring the challenge of extracting clean hemodynamic signals [19]. The interaction between light and biological tissue is highly wavelength-dependent. Ultraviolet light (10–380 nm) is predominantly absorbed by epidermal proteins, while visible (380–760 nm) and near-infrared (760–1300 nm) light penetrates several millimeters into tissue, allowing interrogation of deeper vasculature, with hemoglobin and water being the primary absorbers [25]. Widely adopted in wearable devices, PPG detects blood volume modulations driven by the cardiac cycle, enabling continuous cardiovascular monitoring (e.g., BP estimation) under appropriate calibration and constraints [19]. Recent innovations in flexible electronics have significantly improved PPG performance. Organic light-emitting diodes (OLEDs) [26], polymer LEDs [27], and hybrid inorganic–organic devices [28, 29] offer superior mechanical conformity to the skin, significantly improving signal acquisition stability and reducing motion-induced artifacts [26, 27]. Despite its advantages, the accuracy of PPG-based BP monitoring is compromised by motion artifacts, skin pigmentation, tissue thickness, ambient light interference, and even vasomotor activity [30, 31]. Additionally, PPG measurements are typically taken at peripheral sites (e.g., wrist or finger), which may not fully reflect central blood pressure, potentially limiting clinical precision.

Electrocardiography (ECG) measures the heart's electrical activity through skin electrodes on the chest or limbs (Fig. 2b), capturing characteristic waveforms including the P wave (atrial depolarization), QRS complex (ventricular depolarization), and T wave (ventricular repolarization) [20]. Besides, bioimpedance (BioZ) sensing (Fig. 2c) complements ECG by analyzing tissue electrical properties through applied high-frequency currents to detect arterial pulse-induced impedance variations, blood flow dynamics, and tissue dielectric properties [32, 33]. The performance of these electrical modalities is heavily dependent on the electrode–skin interface. Three primary electrode types are utilized: wet, dry, and non-contact. Wet electrodes (Ag/AgCl with hydrogel) [34] provide excellent initial signal quality and low impedance but suffer from long-term drying, irritancy, and performance degradation [35]. Dry electrodes, fabricated from conductive polymers or metal coatings, offer improved durability and comfort for sustained use, though they can be more prone to motion artifact without hydrogel [36]. Besides, non-contact electrodes, employing capacitive coupling through an insulating layer or elastic electrolytes, represent a significant advancement by eliminating direct skin contact, thereby maximizing user comfort and minimizing irritation and motion artifacts, making them suitable for wearable health monitoring applications [37].

Auscultatory methods based on Korotkoff sounds (K-sounds) represent a classical approach reinvented for modern wearables. These acoustic signals are produced by turbulent blood flow when an artery is partially constricted. Figure 2d reproduces the “core” theory of the mechanism and compares the changes in K-sounds produced by normal and abnormal cardiac function. These sounds are categorized into five phases, with the first (K1) marking the onset of SBP and the fifth (K5) denoting DBP as flow stabilizes [22]. While traditionally detected via stethoscope, modern approaches leverage acoustic sensors and signal processing techniques to capture and analyze these sounds. Recent advances incorporate deep learning to model the complex temporal and spectral patterns of K-sounds [22], demonstrating promise for automated, auscultatory-based BP assessment. Another application of acoustic sensing is the emerging flexible ultrasound technology (Fig. 2f). Leveraging the deep penetration and high spatiotemporal resolution of ultrasound waves, flexible ultrasound sensors have emerged as a powerful tool for non-invasively monitoring central blood pressure, which carries greater clinical significance than peripheral pressure [38, 39]. Acoustic sensors provide more direct physiological measures of pressure and flow, though often at increased cost and system complexity.

Mechano-electric sensors, which transduce mechanical pressure or vibration into quantifiable electrical signals through physical contact, encompass a diverse family including piezoresistive, capacitive, piezoelectric, and triboelectric types (Fig. 2e). Piezoresistive sensors operate on the principle of pressure-induced resistance change. Their performance is enhanced through material innovation (e.g., graphene porous networks [43], carbon nanotube/PDMS composites [44]) and microstructure design (e.g., micropillars, honeycombs), achieving high sensitivity (> 1 kPa⁻^1^), wide dynamic range, and excellent cyclic stability (> 8000 cycles) [45]. Capacitive sensors measure pressure via capacitance changes [42]. Performance optimization focuses on microstructured dielectrics (e.g., micro-pyramids [46] and others [47]) to concentrate stress and reduce modulus and optimized electrode materials (e.g., CNTs, ITO, metal coatings [48]) for flexibility and conductivity. This yields devices with exceptional sensitivity (down to 0.1 Pa), rapid response (~ 10 ms), and low hysteresis, helpful for high-fidelity pulse waveform acquisition [46, 49]. Piezoelectric sensors convert mechanical deformation into electrical charge through intrinsic material polarization [50]. Strategies to boost sensitivity include nanoparticle doping (e.g., BaTiO₃ in PVDF [51, 52]) and microstructuring (pyramids, waves [53]), achieving outputs exceeding 685 mV N^−1^. Ultra-flexible, skin-conformable patches fabricated via techniques like laser lift-off enable stable long-term monitoring [54, 55]. Dynamic analyses have further shown that piezoelectric sensors can faithfully capture arterial pulse waveforms, providing a mechanophysiological link to blood pressure [56]. Triboelectric sensors use contact electrification for self-powered sensing [7]. Nanostructured surfaces (e.g., nanogratings [57, 58]) and textile integration [59] have led to sensitive, comfortable, and robust devices [60, 61]. Moreover, system-level integration with wireless modules and low-power circuits has been achieved, allowing continuous, real-time hemodynamic monitoring in wearable form factors [62, 63], but these sensors still face challenges in static pressure detection and long-term stability.

Radar-based systems, particularly millimeter-wave radar (30–300 GHz), operate by emitting electromagnetic waves and analyzing the phase or frequency shift of signals reflected from the body surface, which vibrates minutely with each cardiac cycle (Fig. 2g). Systems like mmBP [64] employ advanced signal processing and neural networks to extract pulse signals and achieve accurate estimation, reporting deviations of 9.00% for SBP and 3.69% for DBP. Other systems integrate continuous-wave radar with BioZ and ECG to derive pulse arrival time (PAT) or pulse transit time (PTT) for BP estimation, showing strong statistical correlations with reference methods [23, 64]. Radar methods enable unique non-contact operation but are still evolving in terms of accuracy and robustness.

Nevertheless, accurate BP estimation using wearable sensor modalities, such as PPG, ECG, bioimpedance, and tonometry, is challenged by multiple physiological and environmental factors in practice [32, 65], including vasomotor activity (e.g., vasodilation or vasoconstriction), motion artifacts, skin tone variations, temperature and respiratory influences, arterial stiffness, sensor placement inconsistencies, contact-pressure drift, and physiological variability (e.g., heart rate, autonomic activity, blood viscosity) [31]. These factors distort sensor signals, complicating reliable BP measurement, particularly as vasomotor activity can counteract BP-related changes in PPG signals. Independent evaluations have underscored these challenges for commercial cuffless systems in real-world settings [66–68]. Further comprehensive evaluation of continuous BP monitoring sensors and their model will be even more necessary and critical which will be discussed later in this review.

## AI-Based BP Estimation Model

Advancements in wearable sensing technologies have enabled the acquisition of high-quality and varied physiological signals, which has spurred the development of AI-driven models for accurate, non-invasive BP estimation. This section, we will introduce these AI-driven models and their limitations, which are essential for their effective implementation in continuous physiological monitoring.

Current research on AI-based BP estimation models encompasses diverse methodological paradigms, each contributing to distinct aspects of accuracy, interpretability, and adaptability. Broadly, these methods can be categorized into two primary technical routes: physics- or physiology-informed models and machine learning models. Both approaches leverage observable hemodynamic signals and derived parameters such as PPG, ECG, pulse wave velocity (PWV), and pulse transit time (PTT) to estimate BP. However, these surrogate parameters do not directly reflect absolute BP values, necessitating calibration to establish a reliable mapping between the measured parameters and BP [69]. Mathematically, the cuffless BP estimation problem can be formulated as:

1 $$BP= {f}_{\theta }(x,\Phi )$$

where x denotes the input vector derived from physiological measurements, Φ represents subject-specific physiological parameters, *f*_*θ*_ is the mapping function parameterized by θ, the BP includes both beat-to-beat BP values (e.g., SBP and DBP) and the continuous BP waveform, reflecting dynamic hemodynamic changes over time. In the following, we will introduce the state-of-the-art BP methods, and the details of corresponding calibration strategies are referred to Note S1.

### Physics or Physiology Informed Network

Physics- or physiology-informed network (PPIN) incorporates cardiovascular and hemodynamic principles to model the relationship between physiological signals and BP. These models define the BP estimation mechanism through mathematical and biophysical equations rooted in domain knowledge. In this context, *f*_*θ*_ is a predefined function derived from hemodynamic principles or physics laws; *θ* represents universal constants (e.g., blood density, geometric ratios, or fluid constants [70]) that are assumed to be invariant across individuals. In contrast, Φ represents subject-specific calibration parameters (e.g., baseline SBP₀ and DBP₀, reference PTT₀, and vascular elasticity coefficients [71, 72]). Unlike purely black-box AI methods, PPINs offer interpretable, knowledge-driven insights. The foundation of PPINs in blood pressure estimation is related to existing analytical techniques [7, 73], including the arterial BP physiological regulation, the arterial wall mechanics, and the arterial pulse wave propagation model.

**Fig. 3:**
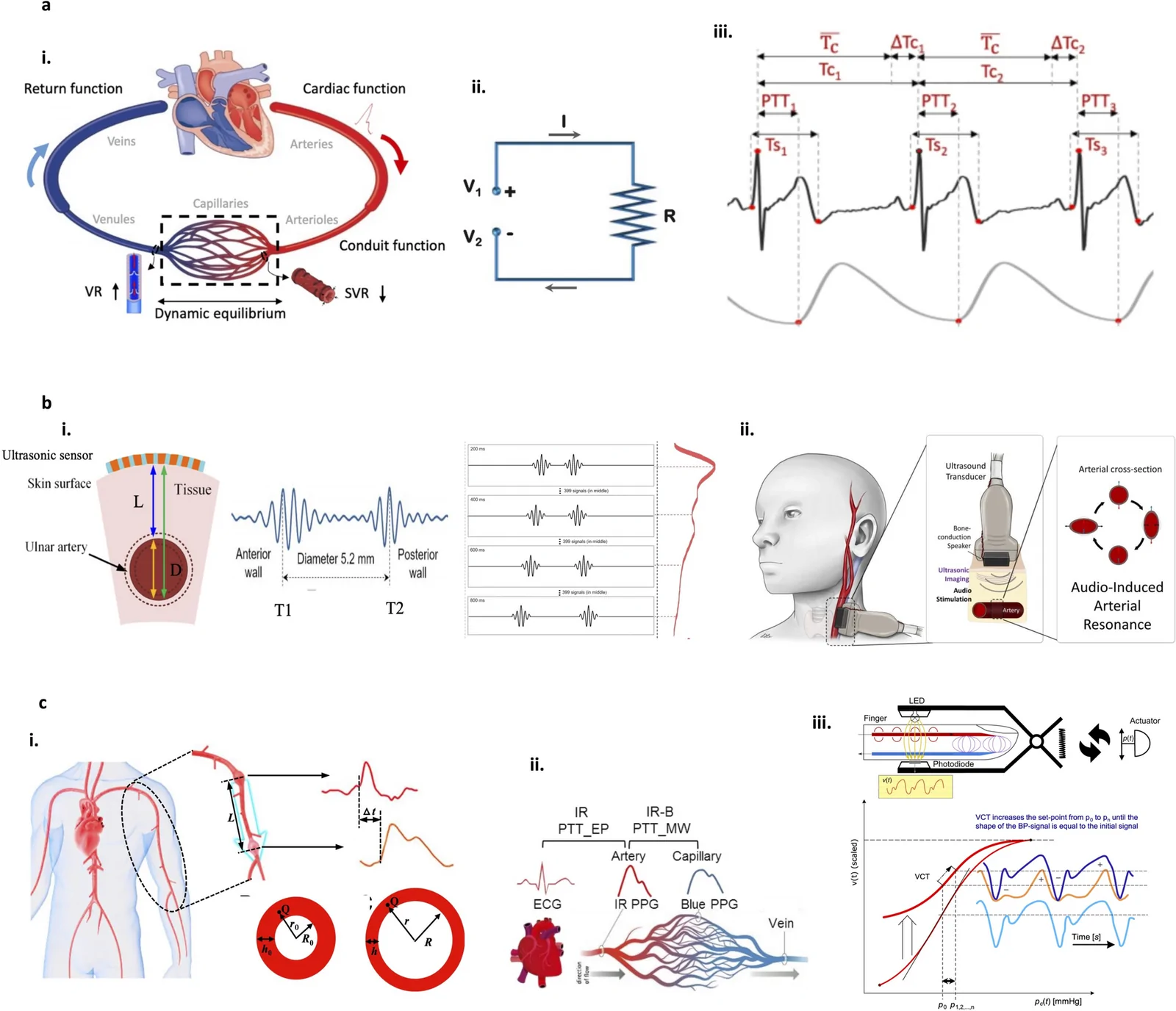
Physiology informed models for cuffless BP estimation. **a** Physiological regulation of arterial BP, influenced by CO, SVR, arterial compliance, and blood volume, modeled through the Windkessel framework. Adapted with permission from [76], Copyright IEEE, 2022. **b** Arterial wall mechanics-based models estimate BP from vessel elasticity or diameter changes, incorporating flexible ultrasound and resonance sonomanometry. i) Adapted with permission from the authors of [102]. ii) Adapted with permission from [84], Copyright Oxford University Press, 2024. **c** Pulse wave propagation models based on PTT, PWV, MWPTT, and vascular control to infer BP with improved robustness under physiological variability. i) Adapted with permission from [89], Copyright IEEE, 2016. ii) Adapted from [83], PNAS, 2019, under the terms of the Creative Commons CC-BY license. iii) Adapted from [94], Nat. Commun., 2021, under the terms of the Creative Commons CC-BY license

The physiological regulation of arterial BP is influenced by arterial compliance, cardiac output (CO), systemic vascular resistance (SVR), and blood volume, Fig. 3a-i&ii. According to the Windkessel model, mean BP (MBP) = CO × SVR [74]. While CO is measurable, SVR is not, complicating BP modeling. Multi-wavelength pulse transit time (MWPTT [75]) and cardiovascular coupling models with heart rate and systolic time interval [76] improve BP estimation accuracy by correlating these parameters to SVR. Typically, these related parameters are determined by physiological signals like ECG and PPG [77], Fig. 3a-iii. In addition, factors such as vascular resistance, the renin–angiotensin–aldosterone system [78], arterial diameter, skin temperature [79], and blood viscosity [80] influence SVR, requiring further quantitative research. Besides, a more complicated cardiovascular hybrid modeling was developed by Shi et al. [81] to directly reconstruct arterial BP waveforms from PPG signals. In practice, the Winkessel model estimates systemic arterial compliance and total peripheral resistance from pulse pressure data, commonly used in cardiovascular research [74]. It uses calibrated arterial pressure waveforms, suitable for real-time monitoring. However, it assumes a lumped parameter system, limiting accuracy for localized pressure dynamics and complex vascular geometries, and requires precise calibration.

Arterial BP can also be estimated from arterial wall mechanics, i.e., arterial elasticity or distensibility, using local PWV or diameter variation [82]. The Hughes equation [83] provides an empirical arterial elasticity-BP link, while the distension-based BP model proposes an exponential relationship, unproven in microcirculation. Flexible ultrasound [34] enables direct vessel diameter measurement (Fig. 3b-i) that posits an exponential relationship between BP and arterial cross-sectional area: $P\left(t\right)={P}_{d}{e}^{\alpha \left(\frac{A\left(t\right)}{{A}_{d}}-1\right)}$, where α is the vessel rigidity coefficient. However, α may vary with daily activities or physiological changes, posing challenges for long-term tracking accuracy without frequent recalibration. Alternatively, resonance sonomanometry [84] (Fig. 3b-ii) offers a calibration-free approach, capturing audio-induced arterial resonance. However, its application requires accurate arterial geometry measurement and has limited validation. These models leverage arterial wall biomechanics (e.g., elasticity, stiffness) to estimate BP via vessel deformation and stress–strain analysis. They are suited for detailed arterial property analysis, such as age-related vascular stiffening or hypertension-induced remodeling studies, relying on imaging modalities (e.g., ultrasound, MRI). While demanding high computational resources and expertise, they are limited by the need for high-resolution imaging and patient-specific data and are sensitive to assumptions about arterial properties.

Arterial pulse wave propagation models are well-known and fundamental to cuffless BP estimation, particularly those using PTT, PAT, or PWV (Fig. 3c). Classical models like Moens–Korteweg and Bramwell–Hill equations [70, 71], which link PWV with BP, are limited by idealized thin shell assumptions [85]. Ma et al. [83] introduced an analytical alternative based on the Fung hyperelastic model (Fig. 3c-i), expressing BP as $P=\alpha {PWV}^{2}+\beta$, avoiding reliance on empirical assumptions, though requiring further validation. Recent studies have improved PTT measurement using signals such as ballistocardiography (BCG) [86], impedance cardiography (ICG) [87], seismocardiography (SCG), phonocardiography (PCG) [88], and multi-wavelength photoplethysmography (MWPPG, Fig. 3c-ii) [75, 89]. Modern extensions incorporate multimodal signal features to increase robustness [72, 90, 91]. Xiang et al. [79, 92] proposed multimodal physiological models integrating temperature, PPG, ECG, and IPG. These methods are suited to non-invasive BP estimation in clinical and wearable settings, especially for PTT or PWV techniques. It uses sensors to capture pulse wave signals (e.g., photoplethysmography, ECG) at multiple arterial locations. While adaptable for continuous monitoring, it needs robust signal processing to mitigate noise. Accuracy relies on precise transit time and distance measurements, which can be compromised by motion artifacts or anatomical differences. Furthermore, it may face challenges with complex wave reflections in impaired arteries.

In contrast to previous pulse analysis techniques, the volume clamp method, also known as the Penaz method [93, 94] (see Fig. 3c-iii), employs advanced vascular control strategies for fingertip pulse monitoring. This approach uses a high-precision controller to apply targeted pressure, maintaining constant vessel volume at the monitoring site to capture an optimal PPG signal. However, the method requires expensive, high-precision controllers and small cuffs to ensure precise pressure regulation and intimate contact with the finger. Additionally, it relies on initial calibration using an oscillometric method to ensure accuracy.

Recently, physics-informed neural networks (PINNs) embed physical laws such as continuity equations and nonlinear partial differential equations (PDEs) into the neural network's training process were developed [95–100], enabling models to learn from data while simultaneously respecting known physiological principles. Originally demonstrated in domains such as fluid mechanics [95] and power systems [96], PINNs are increasingly being applied to cardiovascular modeling. Sel et al. [97] employed a PINN architecture combining a two-layer CNN with bioimpedance signals, incorporating impedance-derived hemodynamic features (e.g., pulse wave velocity, arterial volume) into the model, reducing the requirement for ground-truth training data by a factor of ~ 15. Building upon this, a physics-informed temporal networks (PITN) with temporal blocks and adversarial contrastive learning [98], a DeepONet constrained by the Navier–Stokes equation with time-periodic conditions and Windkessel-type boundary conditions [99], and meta-learning with physics-driven modeling [100], were developed, respectively, to mitigate the interpretation and accuracy of the AI-based BP estimation. These examples illustrate the diversity of physical priors that can be embedded within PINNs. Some models enforce relatively simple hemodynamic relations (e.g., continuity of blood flow or Windkessel-type pressure–flow coupling), while others incorporate more complex formulations such as Navier–Stokes fluid dynamics or pulse wave propagation. Given the complexity of blood pressure regulation, it is imperative to add physiologically informed constraints. Since no single PINN formulation can comprehensively encapsulate the full regulatory spectrum, existing approaches prioritize core equations that are both mechanistically grounded and generalizable across individuals. Thanks to PDEs detailed physiological process, these methods are best suited for advanced research or precision medicine, where complex, patient-specific blood pressure dynamics are modeled using sparse or noisy data while enhancing interpretability [97]. The deployment of PINN requires significant computational resources, large datasets for training, and expertise in machine learning and hemodynamics. It is typically implemented in research-grade systems rather than real-time clinical settings. Most importantly, PINNs also depend on the quality of input data and may overfit if physical constraints are not well-defined [101].

A comparative analysis of their advantages and trade-offs would enhance its utility for guiding model selection and implementations, summarized in Table S1.

### Machine Learning Model

**Fig. 4:**
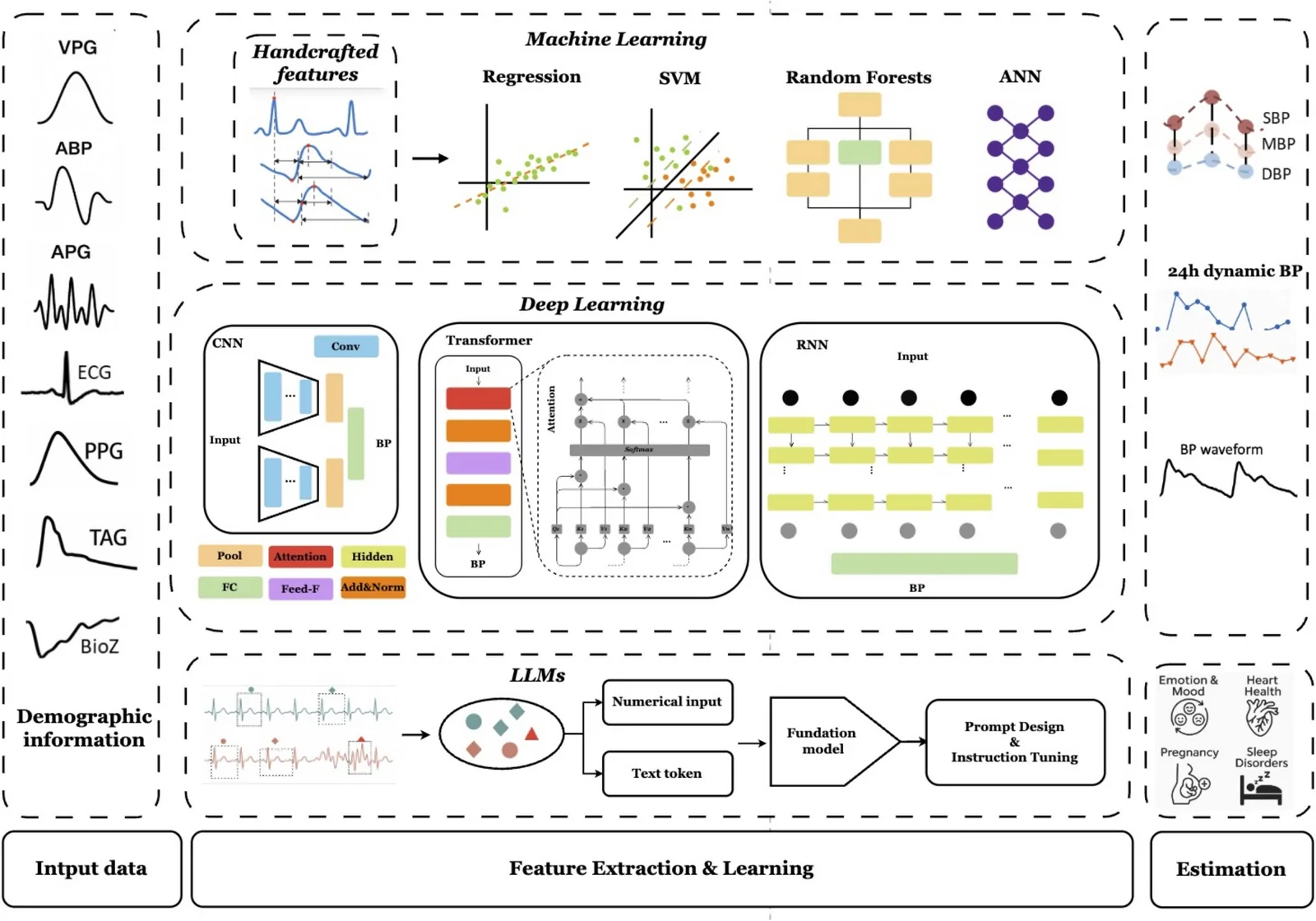
A schematic overview of machine learning-based blood pressure estimation method. Traditional ML methods rely on handcrafted features, whereas DL methods enable end-to-end estimation of beat-to-beat BP values, continuous BP waveforms, and 24-h dynamic BP values from multimodal physiological signals

Machine learning (ML)-based models have contributed significantly to the early development of cuffless BP estimation by capturing complex, nonlinear relationships between physiological signals and BP values without relying on explicit physiological equations. Figure 4 illustrates the flowchart of the process. In this context, *f*_*θ*_ is a trainable function that maps inputs to BP estimations, *θ* denotes model hyperparameters (e.g., layer numbers, neurons count, and activation functions), Φ represents individual-specific or context-related variables (e.g., demographic attributes [103–105]). Through joint optimization of *f*_*θ*_ and Φ, ML models enhance both adaptability and accuracy across heterogeneous populations.

Traditional ML methods typically utilize handcrafted features extracted from signals such as bioimpedance, PPG, or ECG, followed by regression-based models to estimate BP. Representative models include linear regression (LR), multi-instance regression, support vector machines (SVM), ridge regression, random forests (RF), AdaBoost, and artificial neural networks (ANN) [106–108]. While computationally efficient and interpretable, their performance is often limited by feature quality and poor generalizability across subjects and conditions, though they remain useful in data scarcerios.

Deep learning (DL) methods eliminate the need for manual feature engineering by automatically learning spatiotemporal representations from raw physiological signals. CNNs and their variants [109] such as AlexNet [110], MobileNet, EfficientNet, and ResNet capture spatial dependencies and hierarchical features from physiological signals, while Recurrent Neural Networks (RNNs) [111] and their advanced forms including Long Short-Term Memory (LSTM) [112] and Gated Recurrent Units (GRU) [113], are effective in modeling the temporal dependencies of BP-related signals. Hybrid architectures, such as CNN-LSTM [114], CNN-GRU, CNN/RNN-attention [115–117], and Transformer-based models [13, 118], combine the strengths of spatial feature extraction and sequential learning, enhancing model robustness. Temporal Convolutional Networks (TCNs) [119] further improve long-range temporal learning without the need for recurrent connections, making them well-suited for real-time BP monitoring on edge devices.

The emergence of foundation models has introduced a paradigm shift in AI, with growing potential in physiological signal analysis and cuffless BP estimation [12, 120, 121]. These large-scale models leverage massive pretraining data and self-supervised learning to capture generalizable representations across tasks and individuals. PaPaGei [121], the first open-source foundation model for PPG signal analysis, was pre-trained on 57,000 h of data from three public dataset. Its variants, PaPaGei-P and PaPaGei-S, target intra-subject and morphology-based consistency, with PaPaGei-S showing strong performance on cardiovascular tasks including blood pressure, hypertension, and heart rate estimation. SiamQuality [120] uses CNN-based self-supervised architecture to learn quality-invariant representations from over 36 million ICU signal pairs. By pairing high- and low-quality signals, it demonstrates robustness to noise and outperforms conventional baselines on BP estimation and atrial fibrillation detection. Liu et al. [12] explored instruction-tuned Large Language Models (LLMs) for cuffless BP estimation, using ECG and PPG-derived features with domain-specific prompts. Fine-tuned LLMs outperformed task-specific baselines with an estimation error of 0.00 ± 9.25 mmHg for SBP and 1.29 ± 6.37 mmHg for DBP. While foundation models offer notable generalization and adaptability, they still lag behind task-specific models in BP estimation accuracy and impose substantial computational demands.

Table 1 provides a comparative summary of representative studies on BP estimation using PPIN-, ML-, and DL-based methods, detailing input signal types, algorithmic approaches, number of clinically recruited subjects, BP estimation errors, and calibration strategies employed, compensating recent works in standardized benchmarks [122, 123]. These efforts report statistically grounded, cross-dataset results and enable fairer, repeatable comparisons. Nevertheless, the coverage remains incomplete, particularly lacking representativeness among older adults, hypertensive populations, diverse skin tones, and different types of devices. And physics-informed models additionally require standardized evaluations of constraint validity and failure modes. Additional model-level limitations are discussed in Sect. 4.3.

**Table 1:** Comparison of BP estimation methods using physics or physiology informed network (PPIN), machine learning (ML), and deep learning (DL) models

	Input	Method	Datasets (N*)	Evaluation data split*	SBP (mmHg)		DBP (mmHg)		Algorithmic calibration	Test scenario	References
					MAE	ME ± SD	MAE	ME ± SD			
PPIN	MWPTT features	Arteriolar PTT	Private: *N* = 20		1.86	± 2.85	1.49	± 1.75	Personalized	Lab-stress	[75]
	ECG, PPG features	Cardiac-vascular coupling	Private: * N* = 9		3.7	0.07 ± 4.9	4.6	1.4 ± 5.6	Population	Lab-stress, ambulatory	[76]
			MIMIC: * N* = 53		6.8	0.2 ± 8.8	3.8	− 0.1 ± 5.0			
			JOCOC-36 days: * N* = 23		6.2	0.3 ± 8.0	3.7	0.2 ± 4.7			
	Arterial dimensions, artery wall resonances	Resonance sonomanometry	Private: * N* = 6			− 22.7		− 2.1	Calibration-free	Lab-static	[84]
	PTT, PIR features	PIR	Private: * N* = 27		4.09	− 0.37 ± 5.21	3.18	− 0.1 ± 4.1	Personalized	Lab-static	[72]
	PTT features	PTT-IPG	Private: * N* = 15			0.31 ± 8.55		− 0.5 ± 5.1	Personalized	Lab-static	[87]
	ECG, PPG, IPG, temperature features	Multimodal	Private: * N* = 23		5.78	− 1.4 ± 8.0	4.15	− 1.0 ± 5.6	Personalized	Lab-stress	[79]
	IPG signal and features	PINN	Graphene-HGCPT: * N* = 6, calfree-HGCPT: * N* = 5, Ring-CPT: * N* = 4	LOSO*		1.3 ± 7.6		0.6 ± 6.4	Personalized	Lab-static	[97]
	IPG, PPG, millimeter wave signal	PITN	Graphene-HGCPT: * N* = 6, Ring-CPT: * N* = 4, Blumio: * N* = 115	LOSO*		− 0.05 ± 6.1		0.5 ± 5.9	Personalized	Lab-static /stress	[98]
ML	ECG, PPG, PPW features	AdaBoost, SVM	CAS-BP: * N* = 3077	5-folds CV*	7.38	2.3 ± 9.6	5.13	1.3 ± 6.4	Hybrid	Lab-static, ambulatory	[132]
	ECG, PPG	RF	MIMIC-II: * N* = 1212	10-folds CV*	5.2	0.1 ± 6.3	3.8	0.2 ± 4.5	Population	Ambulatory	[133]
	HRV, PPG, PTT features	Adaboost	MIMIC + VitalDB: * N* = 3337	60:20%:20%	7.73	− 0.16 ± 7.96	4.3	− 0.13 ± 4.50	Population	Lab-static	[91]
	ECG, PPW features	Multi-instance regression	Private: * N* = 85	–	6.13	1.62 ± 7.76	4.54	1.49 ± 5.52	Personalized	Ambulatory	[107]
	TAG, PPG, ECG features	Ridge regression	Aurora-BP: * N* = 1125	–		0.32 ± 9.8		0.54 ± 7.7	Population	Ambulatory	[134]
DL	PPG signal	CNN Siamese	MIMIC II: * N* = 304	60%:20%:20%	5.95	± 6.69	3.41	± 3.91	Population	Lab-static	[109]
	PPG signal	CNN-GRU	MIMIC-III: * N* = 50	80%:10%:10%	3.52	0.11 ± 4.56	2.20	0.05 ± 2.82	Hybrid	Lab-static	[135]
	ECG signal	ResNet-LSTM	MIMIC-III: * N* = 1711	65%:10%:25%	7.1	− 0.11 ± 9.99	4.61	0.01 ± 6.29	Population	Lab-static	[114]
	PPG signal	BiLSTM-attention	MIMIC-III: * N* = 225	60%:20%:20%	2.82	− 0.01 ± 4.04	1.88	− 0.13 ± 2.98	Hybrid	Lab-static	[116]
	ECG signal	BiLSTM-attention	MIMIC II: 21,442 records	80%:10%:10%	7.16	0.18 ± 10.8	3.89	1.24 ± 5.90	Hybrid	Lab-static	[117]
	PPG signal	TCN	multi-datasets: * N* = 134	19%:81%	8.9	0.99 ± 7.91	5.8	0.36 ± 5.43	Personalized	Lab-static, ambulatory	[119]
	ECG and PPG features	ANN + RNN	VitalDB: * N* = 1376	70%:30%	5.07	0.05 ± 6.92	2.86	− 0.05 ± 3.99	Population	Lab-static	[111]
	Features and ECG, PPG, PPW signals	CNN-Transformer	CAS-BP: * N* = 1272	60%:20%:20%	6.3	0.7 ± 8.3	5.1	0.9 ± 6.5	Population	Ambulatory	[118]
			Aurora-BP: * N* = 1125		6.1	− 0.4 ± 8.6	5.25	− 0.4 ± 7.0	Population		
	ECG, PPG features	LLaMA3-8B	CAS-BP: * N* = 1272	–	7.08	0.00 ± 9.25	5.31	1.29 ± 6.37	Hybrid	Lab-static	[12]

*N: subject numbers

*LOSO: leave-one-subject-out

*CV: cross-validation

*Data split: the percentage of participants in train:validation:test/train:test dataset

## Challenges and Solutions of BP Deployment in Resource-Limited Settings

The deployment of AI-enhanced BP monitoring in resource-limited settings introduces multifaceted challenges that extend beyond algorithmic accuracy. Limitations in computational hardware, power supply, data availability, and healthcare infrastructure demand a holistic rethinking of system architectures and deployment paradigms. To achieve clinically viable, scalable, and accessible implementation, it is essential to integrate model efficiency with hardware feasibility and real-world usability. This section systematically reviews representative computing architectures, resource-aware optimization strategies and analyzes persistent barriers and emerging solutions shaping the field.

### Resource-Aware Computing Architectures

Deployment of AI models under resource constraints requires computing architectures that align model complexity with hardware capabilities [124, 125]. Table 2 summarizes representative academic efforts in deploying AI-based BP estimation, detailing input modalities, optimization techniques, hardware platforms, and performance metrics (e.g., latency, memory, energy, and clinical accuracy). All methods in Table 2 were validated in laboratory scenarios [68], including lab–static (controlled resting), lab–stress (acute perturbations such as cold pressor, posture/handgrip, pharmacologic stimuli). In parallel, Table 3 catalogs commercial cuffless BP systems (e.g., Aktiia, Omron HeartGuide, Valencell) from peer-reviewed publications, FDA filings, and manufacturer whitepapers. This consolidated comparison facilitates cross-method evaluation and informs practical design decisions under real-world constraints.

**Table 2:** Overview of the representative studies on the deployment of cuffless BP estimation model

Input	Sampling rate and windows	Models	SBP (mmHg)		DBP (mmHg)		Deployment device	Run time	Parameters	Memory	Power consumption	References
			MAE	ME ± SD	MAE	ME ± SD						
4 ECG, PPG features	-, 8 s	LR	16.70		7.59		Arduino uno, ESP32, PyBadge	µs level		< 10 KB		[136]
		SVM	16.92		7.38			µs level		< 10 KB		
		DT	14.49		6.98			~ 15–40 µs		10 ~ 50 KB		
		RF	14.08		6.85			> 50 µs		> 100 KB		
rPPG	25 Hz, 12 s	ResNet-attention	9.27		5.84		Intel i7	1.616 s	~ 2.4 M			[137]
PPG features	125 Hz, beats	XGBoost	7.27	± 9.5	3.33	± 4.55	24-cores CPU	~ 2.36 ms		49.55 KB		[138]
PPG signal	125 Hz, 2.1 s/5 s	ResNet, UNet	17.2		8.08		GAP8	7.04–8.91 ms	23.4 ~ 156.3 k	< 512 KB	0.36 ~ 0.45 mJ	[139]
PPG signal	125 Hz, beats	ANN	3.42	± 5.42	1.92	± 3.30	EFM32	42.2 ms		< 25 KB	2.1 mJ	[140]
PPG signal	125 Hz, 10 s	UNet	5.16		2.89		Raspberry Pi 4	42.53 ms				[141]
ECG signal	125 Hz, 4 s	ANN	5.98	0.83 ± 9.13	3.53	0.1 ± 6.2	Intel i7	0.898 s		7 KB		[142]
82 PPG features	125 Hz, 8 s	CNN-BiLSTM	1.38		0.95		Jetson Nano, AGX Xavier					[143]
IPG, BCG signals	500 Hz, 2.048 s	XGBoost	8.58	2.2 ± 10.9	5.27	1.9 ± 6.8	stm32F756ZG	~ ms		339.77 ~ 350.3 KB		[144]
ECG	128 Hz, 0.78 s	NARX-ANN					Jetson Nano	81.8 ms			2000 mw	[128]
PPG signal		LeNet	11.27	2.5 ± 16.9	5.95	1.9 ± 9.6	Raspberry Pi	8 ms	0.30 M	70 KB		[145]
		ResNet		0.6 ± 16.5		2.3 ± 8.7		186 ms	0.27 M			
	125 Hz, 4 s	SqueezeNet		2.5 ± 17.2		2.7 ± 9.0		25 ms	0.80 M			
		AlexNet		− 2.8 ± 14.5		− 2.7 ± 7.8		27 ms	0.94 M			
		MobileNet		− 2.2 ± 17.6		2.2 ± 9.7		12 ms	0.82 M			
PPG features	125 Hz, 3 s	TCN	2.38	0.07 ± 3.2	1.23	0.1 ± 1.7	Raspberry Pi zero w	2.5 s		32.22 KB		[146]
PPG signal	100 Hz, 10 s	SCIGTCN	8.9	0.99 ± 7.9	5.8	0.4 ± 5.4	Jetson Nano	1.92 s				[119]
4 PPG features	125 Hz, beats	CAE	2.25	± 2.82	5.01	± 2.1	Arduino nano 33 BLE					[147]
PPG signal	125 Hz, 0.8 s	ANN	3.85	± 4.29			stm32L4 +		~ 25 k			[148]
ECG, PPG features	125 Hz, −	LSTM	MSE = 0.0151				ASIC	0.215 ms	~ 270 k		3.27 mW	[149]
PPG features	−, 0.4 ~ 3 s	DNN	4.9	± 6.2	3.4	± 4.4	ZYNQ7020	0.3 ~ 4 s	~ 1 k		4.4 uW	[150]
PPG features	−, beats	ANN	2.47	± 3.48	1.45	± 1.88	ASIC				19.72 uW, 0.2 mJ	[151]

**Table 3:** Overview of the representative commercial cuffless BP systems

Device	Input	Method	SBP errors*	DBP errors*	Clearance	Calibration	References
Aktiia	PPG signal	PWA	1.3 ± 7.11	− 0.2 ± 5.46	FDA/CE	Monthly cuff	[152]
Omron HeartGuide	Oscillometric	Oscillometry	− 0.9 ± 7.6	− 1.1 ± 6.1	FDA	No	[153]
Valencell	PPG, demographics	ML	0.0 ± 7.9	0.4 ± 7.4	–	No	[154]
Healthstats	Radial pressure	Tonometry	< 5 ± 8	< 5 ± 8	FDA	Weekly cuff	
Sotera Visi Mobile	ECG, PPG	PAT/PTT	− -1.88 ± 6.17	− 1.65 ± 3.62	FDA	Initial cuff BP, recalibrate ≥ 24 h	
Biobeat BB-613WP	PPG	PWTT	− 0.1 ± 3.6	0 ± 3.5	FDA	Periodic cuff	[155]
LiveOne	Pressure/tonometry	ML	0.0 ± 6.9	1.2 ± 5.7	FDA	Demographic	[156]
NanoWear SimpleSense	PPG, ECG, heart sound, thoracic impedance, activity, demographics	Ensemble ML	− 2.94 ± 4.82	− 0.77 ± 3.75	FDA	Initial cuff BP, periodic update	[157, 158]
PyrAmes/Boppli	Capacitive sensor array	ML	− 0.7 ± 7.7	1.4 ± 4.7	FDA	Demographic	[159]
Biospectal/OptiBP	camera PPG	ML	1.5 ± 6.7	− 0.2 ± 4.1	CE	Cuff	[160]
Samsung Galaxywatch	PPG	PWA	− 2.05 ± 15.5	− 5.58 ± 22.5	CE	Every 28 days cuff	[161]
CART-I	PPG	PWA	1.74 ± 6.69	− 3.24 ± 6.51	Korea MFDS	Two-step cuff (+ periodic)	[162]

*Accuracy metric: ME ± SD (mmHg)

Based on Tables 2 and S2, the deployability can be directly shaped by model-side factors: input configuration (sampling rate, window length, channels), model architecture (depth, width, parameters), target metrics (accuracy, latency, robustness) and optimizations; hardware-side constraints: on-chip SRAM/Flash, compute throughput, and accelerator availability and AFE design.

**Fig. 5:**
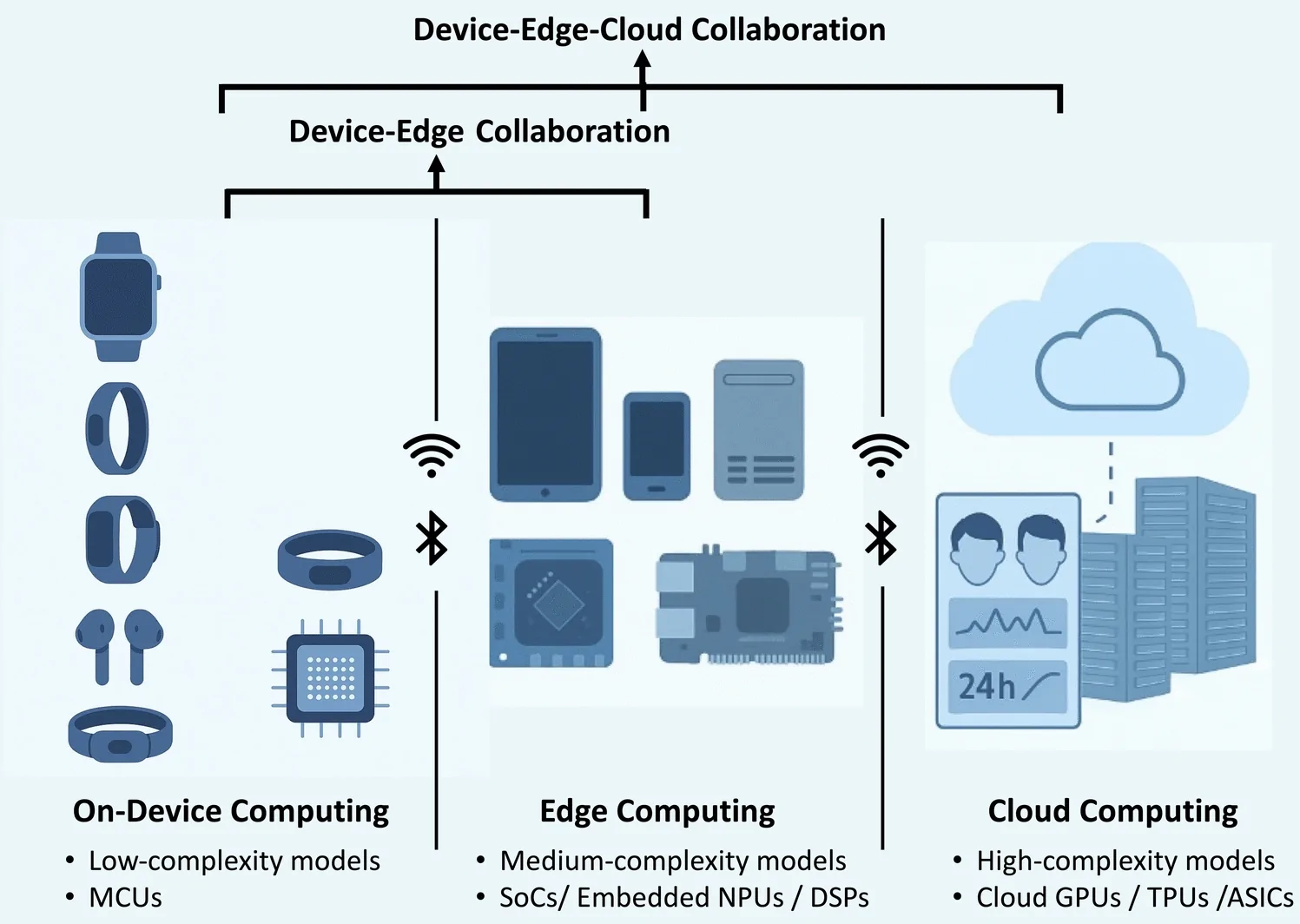
Scalable resource-aware computing architectures for wearable BP monitoring. Three computing paradigms aligned with model complexity and system capabilities: on-device, edge, and cloud

As illustrated in Fig. 5, such deployments are typically structured into these tiers: on-device, edge, and cloud, facilitating adaptive distribution of computational tasks across hardware platforms with differing resource constraints [17, 126]. Accordingly, low-complexity models (e.g., physiology-informed and traditional ML models) align with an on-device tier where sensing, preprocessing, and inference run locally on MCUs or ultra-low-power SoCs (e.g., Nordic nRF52840, ESP32-S3, and STM32N6) for maximal privacy and minimal latency/energy [124, 127]. Mid-complexity models (e.g., compact deep networks) align with a device–edge tier where sensing and light preprocessing remain on the device, while the main inference runs on nearby NPUs/DSPs (smartphones/gateways), balancing responsiveness, and compute [125]. High complexity (e.g., transformers and foundation models) align with a device–edge–cloud tier that keeps local preprocessing and lightweight inference while offloading heavy inference or training to the cloud, trading connectivity, and latency for scalability [128]. Detailed deployment factors and the model–hardware selection strategy are provided in Note S2.

Together, these deployment strategies define a scalable design spectrum for BP monitoring from ultra-low-power real-time inference to cloud-based precision analytics. Robust deployment in resource-limited settings requires coordinated optimization across the system stack, including AFE design, digital processing platforms, and AI model architecture [127, 129–131]. Representative processor platforms and associated trade-offs are summarized in Table S2, with detailed implementation examples provided in Note S3.

### Optimization and Deployment Strategies for BP Estimation

**Fig. 6:**
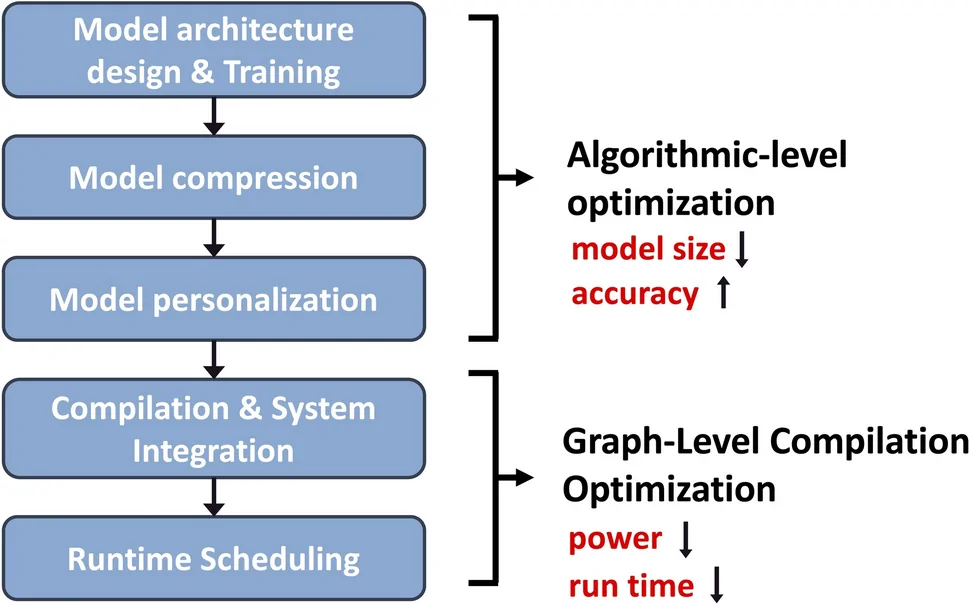
End-to-end optimization pipeline for deploying AI-driven BP estimation on resource-limited devices. The process spans algorithm-level design, compression and personalization, followed by graph-level compilation, system integration, and runtime scheduling

Building upon the computing architectures described above, effective deployment of AI-driven BP estimation models in resource-limited settings requires a structured optimization pipeline that spans both algorithm-level design and graph-level compilation (Fig. 6). This dual-layer pipeline enables scalable execution across heterogeneous platforms from ultra-low-power wearables to edge and cloud infrastructures.

#### Algorithm-Level Optimization

At the algorithm level, model architecture and parameter design must account for memory, energy, and latency constraints while preserving estimation accuracy. Lightweight manually designed models [141, 145, 149] (e.g., MobileNet, SqueezeNet) and hardware-aware Neural Architecture Search (NAS)[139, 163] methods can generate compact models that fit on constrained devices. A typical NAS framework comprises three core components [163]: a search space defining possible architectures and hyperparameters (e.g., layer type, kernel size, depth, width); a search algorithm (e.g., reinforcement learning, evolutionary methods); and a performance estimator that evaluates candidates using full training or proxy methods like weight sharing. The models can be prepared through dynamic graph frameworks (e.g., PyTorch, TensorFlow, PaddlePaddle); they are commonly pretrained on large-scale datasets such as MIMIC-II/III/IV [140–142, 145, 146, 148, 151] and fine-tuned on edge-deployable datasets [119, 136]. Signal-level optimization such as adaptive windowing, downsampling, filtering, and dimensionality reduction (e.g., PCA) further reduce computational load. This stage defines the foundational neural architecture and pretrained weights to improve convergence and generalization. These efforts ensure reliable signal-to-model mappings as a basis for further optimization.

Compression techniques further reduce model size and computational cost. These strategies can be applied synergistically [127, 136] and tailored to target hardware constraints via automated frameworks such as AdaDeep [131].

Pruning reduces model complexity by eliminating redundant weights or structural components (e.g., neurons, channels) [138]. Unstructured pruning (e.g., magnitude-based) yields sparse weight matrices, while structured pruning targets high-level computational units (e.g., neurons, channels, kernels, or entire blocks), yielding a regular architecture optimized for hardware acceleration and parallelism [164]. Energy-aware pruning [165] selectively removes components based on energy cost metrics, enhancing overall power efficiency.

Quantization reduces numerical precision (e.g., FP32 to INT8) for compatibility with integer-only compute engines such as MCUs or NPUs [147, 151]. The widely supported frameworks include TFLite, CMSIS-NN, TensorRT, and QNNPACK.

Knowledge Distillation (KD) transfers knowledge from large “teacher” models to smaller “student” models through various strategies [166]: response-based, where the student mimics the teacher’s soft outputs; feature-based, which transfers intermediate representations (e.g., hidden states); relation-based, capturing inter-sample or inter-layer dependencies.

Low-Rank Factorization approximates large weight matrices with rank-decomposed components, enabling faster inference and model simplification with minimal retraining [167]. It is particularly effective when layers exhibit strong linear dependencies. Common techniques include canonical polyadic decomposition and tucker decomposition [168], where convolutional layers were successfully factorized and fine-tuned to maintain model performance.

Model Personalization enhances real-world robustness by adapting models to individual variability [76, 138]. Personalized adaptation strategies such as transfer learning, feature sharing, and parameter-efficient fine-tuning (e.g., Low-Rank Adaptation (LoRA), QLoRA) were widely used [116, 119, 169]. Continual and incremental learning [149, 170] further support long-term adaptation by updating model parameters in real time, mitigating performance drift without full retraining. In multi-user scenarios, federated learning enables decentralized model updates without raw data exchange, preserving privacy [171]. While not yet widely adopted for BP estimation, split learning offers promising potential for ultra-constrained settings by enabling partial computation offloading to edge servers [172], balancing local adaptation and efficiency. These strategies allow the same model backbone to generalize across users while adapting to temporal or population-specific physiological differences.

#### Graph-Level Compilation Optimization

Once model architecture and parameters are finalized, the trained models are compiled into static computation graphs (e.g., ONNX, TFLite, PaddlePaddle IR), which are then lowered into hardware-executable code through compilation toolchains (e.g., TVM, CMSIS-NN, or TensorRT) [173] with graph- and tensor-level optimizations [130]. Core graph-level optimizations include graph simplification (e.g., constant folding, operator fusion, layout transformation), tensor scheduling (e.g., tiling, unrolling, vectorization) [174], memory planning for buffer reuse and alignment, and auto-tuning for device-specific kernel scheduling. These optimizations maximize efficiency across diverse hardware in BP estimation field, including MCUs, NPUs, and AI ASICs [140, 144, 150].

Compiled binaries are integrated into firmware stacks alongside drivers and real-time operating systems (RTOS) kernels, enabling efficient on-device inference. At runtime, lightweight execution engines (e.g., TFLite Micro, CMSIS-NN) manage memory and task scheduling under RTOSs or bare-metal conditions [175]. Dynamic scheduling techniques [176] have been introduced to enable dynamic adaptation to context, improving responsiveness and energy efficiency in daily use. For instance, dynamic routing selectively activates sub-networks based on input complexity [126], while early exit [125] architectures terminate inference once confidence thresholds are met. Intermittent scheduling enables periodic or event-driven inference aligned with physiological rhythms[146, 150]. In addition to purely on-device execution, hybrid and offload scheduling schemes [17, 125] can alleviate local computational burdens by distributing inference across edge servers or the cloud. In such designs, some researches [128, 137, 177] conduct lightweight signal preprocessing on-device, while offloaded heavier computation tasks.

### Infrastructure, Model, and Deployment Barriers

AI-driven wearable BP monitoring in resource-limited settings faces complex and interdependent barriers spanning infrastructure, model design, practical deployment, and ethical considerations, which limit clinical translation and real-world reliability.

Infrastructure barriers are foundational. Low-cost, energy-constrained wearable devices, and basic smartphones severely limit computational capacity, memory, and data storage [16, 178]. These limitations hinder real-time inference, long-term logging, and scalable data integration. Unreliable power supply and intermittent network connectivity further complicate deployment, making edge-only, or edge-first AI processing essential [179]. Moreover, the lack of standardized data ecosystems and interoperability across heterogeneous devices further hinders scalable and coordinated BP monitoring efforts [14].

Model-level barriers present a critical bottleneck. First, physiological non-specificity remains a foundational barrier: Hemodynamic features extracted from non-invasive signals (e.g., PPG, mechano-electric) are modulated by vasomotor tone, autonomic state, contact pressure, motion, temperature, and device/subject identifiers, leading to feature shifts that are weakly or non-uniquely associated with BP [11]. Second, the burden of calibration and drift is substantial: Many systems require frequent recalibration, and calibration-heavy designs often perform similarly to strong non-physiological baselines (e.g., calibration BP and time). Third, evaluation and implementation flaws, over-optimistic results due to data leakage, hyperparameter tuning on test sets, calibration leakage, and selective metrics. Fourth, subgroup fairness and robustness, small, cohort-biased datasets, and weak generalization protocols limit robustness across real-world conditions such as ambulatory, exercise, and thermal variability [6]. Performance disparities across subgroups (e.g., elderly, hypertensive, different skin tones, or arterial stiffness levels) remain under-explored, posing fairness concerns. Lastly, the absence of prospective, real-time clinical trials prevents regulatory adoption and obscures the actual benefit of cuffless BP systems in long-term management. Early solutions using hardware-aware neural architecture search, model quantization, federated learning, and lightweight personalization show promise [178].

Deployment barriers include energy constraints, maintenance challenges, user trust, and the absence of validation in daily life. In LMICs deployment scenarios, power and hardware constraints must be explicitly aligned with reporting requirements. Clinical applications range from beat-to-beat estimation in critical care, to intermittent 30–60-min monitoring in ambulatory management, to daily or weekly tracking for lifestyle support. These scenarios entail distinct trade-offs in sensing complexity, model latency, and energy consumption. Table S3 contextualizes these trade-offs, linking use cases to deployment constraints and guiding resource-aware system design. Continuous health monitoring imposes substantial energy demands, while reliable charging remains impractical in many resource-limited settings due to unstable electricity infrastructure [14]. Devices must be sturdy, intuitive, and require minimal maintenance, as high device loss and limited local support can critically limit adoption [11]. Acceptance studies have highlighted user concerns around data accuracy, trust, and loss of human interaction, emphasizing the need for transparency and human–AI collaboration [180].

Beyond technical constraints, ethical and regulatory barriers remain. Insufficient transparency, algorithmic bias, data privacy issues, and lack of governance infrastructure continue to limit trust and equitable access in many regions [6, 180]. Integrative deployment strategies combining edge inference with human-in-the-loop oversight and community health can alleviate trust barriers and enhance usability [10].

Despite these challenges, encouraging precedents from adjacent domains demonstrate that meaningful clinical impact is possible through thoughtful system-level design. Wearable systems have shown real-world utility in fetal and maternal monitoring, arrhythmia detection, and hearing screening when paired with frugal hardware and edge-optimized AI [15, 16, 179]. For instance, Ryu et al. developed a wireless network of soft, flexible sensors capable of comprehensive maternal and fetal monitoring including HR, uterine activity, and fetal movements across both high- and low-resource settings [15]. Their system was validated in clinical environments ranging from tertiary hospitals to rural clinics, demonstrating the feasibility of low-cost, wearable-based monitoring even in infrastructure-limited environments. Similarly, Chan et al. presented an off-the-shelf otoacoustic emission probe using low-cost hardware and AI-based signal interpretation to enable newborn hearing screening in LMICs [16]. This study illustrates how combining frugal hardware design with intelligent signal processing can make clinical-quality screening accessible at scale. Recent work demonstrated that AI models optimized for consumer smartwatches can achieve clinical-grade performance in detecting critical physiological events such as loss of pulse, with fully edge-based, energy-efficient inference [179]. These successes may offer valuable design patterns translatable to cuffless BP monitoring. At the infrastructure level, low-cost sensing and edge AI reduce reliance on cloud connectivity and power-hungry processing.

## Evaluation and Validation

### Model Evaluation Metrics

Accurate evaluation is critical to ensure that AI-based cuffless BP estimation systems are clinically valid and practically deployable [40]. Researchers usually use a variety of metrics to evaluate model performance against reference methods (e.g., intra-arterial or auscultatory cuff-based measurements), including mean error (ME), standard deviation of error (SDE), mean absolute error (MAE), root mean squared error (RMSE), and mean absolute percentage error (MAPE) [181] for waveform-based methods. Tracking metrics such as time-series RMSE or mean tracking error are employed to assess a model’s responsiveness to BP fluctuations in dynamic monitoring scenarios. Correlation coefficient (e.g., Pearson’s r) and Bland–Altman plots evaluate trend alignment and agreement with reference values.

In addition, recent studies have advocated more rigorous and reproducible practices that directly probe physiological discriminability, calibration dependence, and real-world robustness: (1) Leakage-free evaluation, using subject-wise splits to prevent information leakage from the same individual. (2) Baseline comparisons against non-physiological models (e.g., models rely solely on non-measurement features such as calibration BP, population-average BP, demographics, or time of day) [12, 182] to assess physiological signal utility. In the Microsoft Research Aurora Project [67], all models based on waveform features produced errors comparable to those of a baseline model using only calibration BP and time. In parallel, include cuff-anchored baselines (e.g., periodic upper-arm cuff recalibration) to assess any added benefit beyond calibration. (3) Feature attribution (e.g., explainable methods such as SHAP [65]) and ablation studies are recommended to quantify the contribution of input features to the final estimations. (4) BP changes (ΔBP) tracking under induced BP changes (e.g., physical exercise, vasomotor provocation tasks such as cold pressor tests, mental stress tasks, or pharmacological interventions) and over long time to assess dynamic adaptability. (5) Individual-level evaluation, identifying error distributions across subgroups (e.g., elderly, hypertensive, different skin tones/arterial stiffness), addressing fairness and robustness. (6) Lab-to-ambulatory with concurrent logging of motion, skin temperature, contact pressure, posture, and other contextual variables, to assess degradation in non-ideal settings. (7) Reporting and metrics, explicitly document calibration burden and schedules, and release protocols/device settings/code where possible to enable reproducibility.

### On-Device Evaluation

In addition to model robustness and fairness evaluations, on-device evaluation focuses on assessing the system-level performance and robustness of BP estimation models when deployed on resource-limited wearable platforms. Key system-level metrics [17, 127, 136, 139, 145] include model size, inference latency (ms), floating point of operations (FLOPs), memory footprint (KB), energy consumption (mJ), and clinical accuracy which are assessed to ensure that the system meets real-world requirements under diverse operating conditions. Comparisons between edge and server inference typically show tolerable accuracy degradation (~ 8%–10%), confirming the feasibility of wearable BP estimation. Beyond these core metrics, robust on-device evaluation should further encompass assessments of system robustness and long-term usability. This includes: (1) Robustness to motion artifacts and environmental noise [7], by testing model performance under controlled motion scenarios (e.g., walking, wrist rotation) and across varying ambient conditions (light, temperature, humidity), using both synthetic and real-world datasets. (2) Battery impact analysis [183], quantifying the additional power consumption introduced by BP estimation tasks, and evaluating its effect on overall device battery life under typical usage patterns. 3) OTA update robustness, validating the integrity and consistency of model performance following over-the-air updates, ensuring clinical reliability is maintained post-update.

Finally, the lack of standardized benchmark datasets and testing protocols for on-device BP estimation poses a challenge to cross-study comparisons. Establishing such benchmarks—including standardized motion protocols, battery stress tests, and runtime performance evaluation guidelines—would greatly enhance the comparability of published results and accelerate progress toward clinically robust wearable BP monitoring systems.

### Standard Requirements

**Fig. 7:**
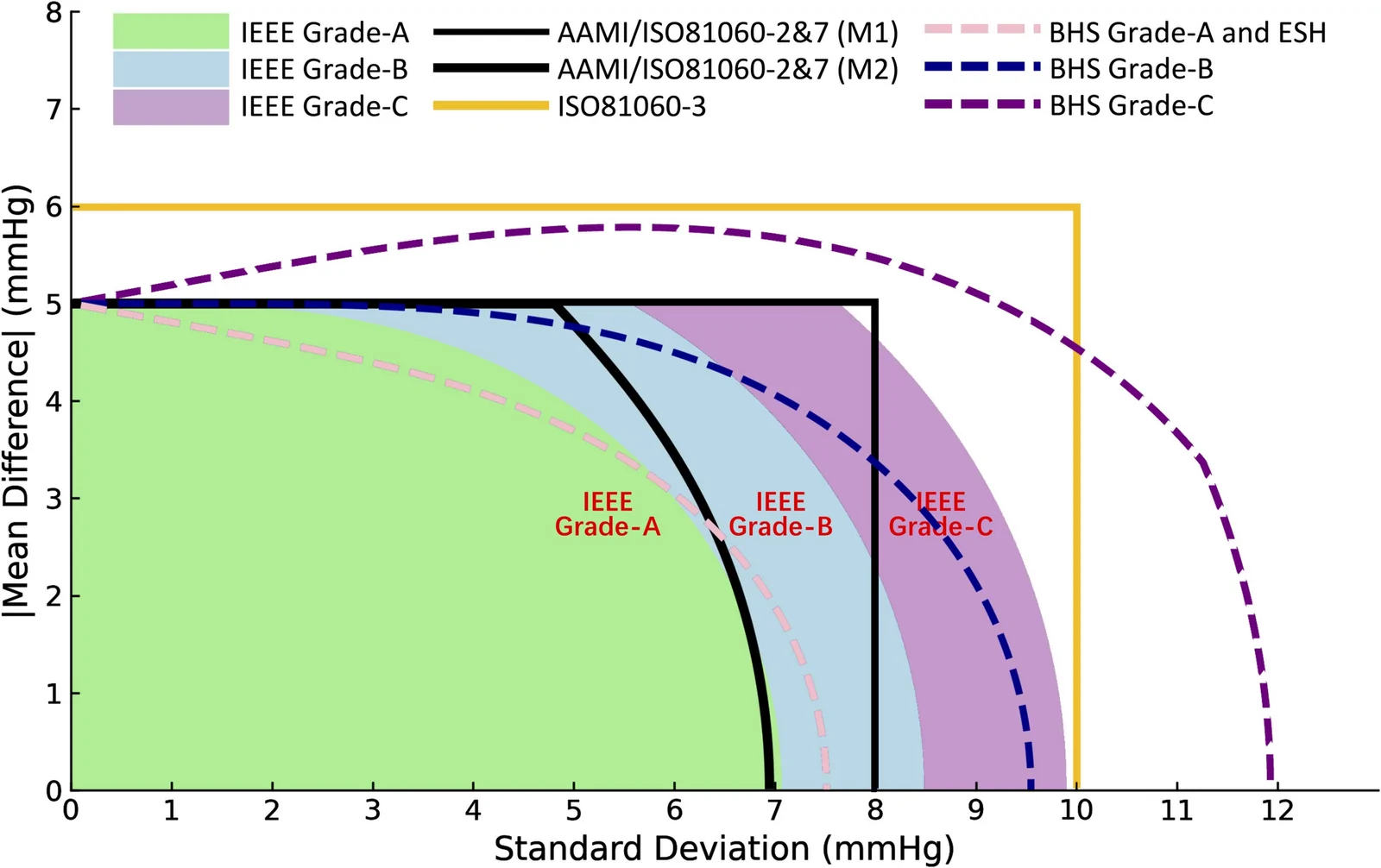
Accuracy grading criteria for different standards. Adapted with permission from [92], Copyright IEEE, 2025. The x-axis represents the standard deviation of error, and the y-axis denotes the mean error. The shaded regions indicate the accuracy grading criteria defined by the IEEE 1708-2014/2019 standard. The solid line corresponds to the clinical thresholds specified by the AAMI/ESH/ISO standard, while the dashed lines represent the grading levels defined by the BHS protocol

To enable clinical translation, cuffless BP estimation methods must align with internationally recognized standards, many of which were initially developed for cuff-based systems but are now widely referenced for wearable and cuffless technologies. These standards define acceptable error thresholds, data distribution requirements, validation protocols, and reference measurement methods. The AAMI/ESH/ISO standard [184] mandates static testing with normal data distribution and fixed thresholds, along with a demographically diverse (e.g., age, gender, arm circumference) subject pool. The British Hypertension Society (BHS) standard [185] provides a grading system (A–D) based on the cumulative percentage of errors within 5, 10, and 15 mmHg (CP5, CP10, CP15), requiring independent accuracy for both SBP and DBP. The IEEE 1708-2014/2019 standards were the first tailored for ‘cuffless wearable BP devices’ [42, 46], which introduce the use of mean absolute difference (MAD) and mean absolute percentage difference (MAPD) as key metrics and emphasized dynamic testing (e.g., postural and motion). The ISO 81060-3:2022 standard, targeting continuous automated sphygmomanometers, adopts a relaxed criterion with simplified requirements [186]. More recently, the European Society of Hypertension (ESH) [103] released an application-driven protocol specifically for cuffless BP systems, incorporating six evaluation scenarios and advanced statistical modeling (e.g., mixture of multivariate normal distributions). These standards provide essential benchmarks for assessing model performance, guiding validation efforts, and determining clinical acceptability. The specific metrics and performance thresholds summarized in Table 4 and the grading framework [92] illustrated in Fig. 7 offer a comprehensive overview of the prevailing regulatory landscape.

**Table 4:** Summary of BP validation standards

Standard	Sample size	Reference method	Acceptance criteria
AAMI/ESH/ISO	≥ 85	Sphygmomanometer/ invasive arterial line	ME ≤ ± 5 mmHg, SD ≤ 8 mmHg
BHS	85	Sphygmomanometer	Grade A: CP_5_ ≥ 60%, CP_10_ ≥ 85%, CP_15_ ≥ 95% Grade B: CP_5_ ≥ 50%, CP_10_ ≥ 75%, CP_15_ ≥ 90% Grade C: CP_5_ ≥ 40%, CP_10_ ≥ 65%, CP_15_ ≥ 85% Grade D: Worth than Grade C
IEEE 1708-2014/2019	≥ 85	Sphygmomanometer/ invasive arterial line	Phase 1: MAD ≤ 7 mmHg Phase 2: Grade A: MAD ≤ 5 mmHg Grade B: 5 < MAD ≤ 6 mmHg Grade C: 6 < MAD ≤ 7 mmHg Grade D: MAD > 7 mmHg
ESH 2023	85–175	Auscultatory/24-h oscillometric	ME ≤ ± 5 mmHg, SD ≤ 8 mmHg
ISO	85	Invasive arterial line	|ME|≤ 6 mmHg, SD ≤ 10 mmHg

## Summary

The integration of non-invasive sensing, artificial intelligence, and resource-efficient deployment offers transformative potential for cardiovascular health monitoring, particularly in blood pressure management in low- and middle-income countries. Wearable AI-driven BP monitoring systems show promise for hypertension screening and long-term care, though current cuffless wearables are best suited as trend-tracking companions. Key challenges include developing high-fidelity, low-cost, and energy-efficient sensors, lightweight, low-power models that can accurately distinguish BP changes from confounding factors, and addressing the issue of frequent calibration. Furthermore, leveraging regional resources to enable scalable deployment of BP models is crucial. Data scarcity, lack of standardized datasets, and the need for robust evaluation batteries also impede progress. Clinical deployment faces challenges related to regulation, ethics, and trust, necessitating standardized validation frameworks and independent assessments. Future solutions should be designed for global impact, prioritizing frugality, resilience, interpretability, and clinical meaningfulness to democratize cardiovascular diagnostics and support accessible care.

## Supplementary Information

Below is the link to the electronic supplementary material.

Supplementary file1 (DOCX 77 kb)
