# Supplementary material for: Artificial Intelligence-Enhanced Wearable Blood Pressure Monitoring in Resource-Limited Settings: A Co-Design of Sensors, Model, and Deployment
Source: Nanomicro Lett. 2026 Jan 5;18:164. doi: 10.1007/s40820-025-02003-9 (PMC12765800; doi:10.1007/s40820-025-02003-9)
Supplement: Supplementary file 1 — Supplementary file1 (DOCX 77 kb) [file 40820_2025_2003_MOESM1_ESM.docx]

Supporting Information for

**Artificial Intelligence-Enhanced Wearable Blood Pressure Monitoring in Resource-Limited Settings: A Co-Design of Sensors, Model, and Deployment**

Yiming Zhang^1,#,^*, Shirong Qiu^1,#,^*, Kai Du^2^, Shun Wu^1^, Ting Xiang^3^, Kenghao Zheng^1^, Zijun Liu^3^, Hanjie Chen^1^, Ji Nan^1^, Fa Wang^4^, Weijia Wu^5^, and Yuan-Ting Zhang^1,^*

^1^ Department of Electronic Engineering, The Chinese University of Hong Kong, Hong Kong SAR 999077, P. R. China

^2^ College of Electronic and Information Engineering, Southwest University, Chongqing 400715, P. R. China

^3^ Department of Biomedical Engineering, City University of Hong Kong and Hong Kong Centre for Cerebro-Cardiovascular Health Engineering (COCHE), Hong Kong SAR 999077, P. R. China

^4^ United imaging microelectronics technology, Shanghai 201815, P. R. China

^5^ Department of Electrical & Computer Engineering, National University of Singapore, Singapore

^#^ Yiming Zhang and Shirong Qiu contributed equally to this work.

## *Corresponding authors. E-mail: [yimingzhang@cuhk.edu.hk](mailto:yimingzhang@cuhk.edu.hk) (Yiming Zhang); [srqiu@link.cuhk.edu.hk](mailto:srqiu@link.cuhk.edu.hk) (Shirong Qiu); [ytzhang@cuhk.edu.hk](mailto:ytzhang@cuhk.edu.hk) (Yuan-Ting Zhang)

## Note S1 Calibration Strategies

1. **Modeling Paradigms and Calibration Targets**

Calibration is essential due to the indirect nature of cuffless BP measurements and the inherent complexity of human physiology. Variability introduced by factors such as vascular stiffness, diet, physical activity, and inter-individual differences can significantly affect estimation accuracy. Calibration refers to the process of refining model predictions to align with individual-specific BP measurements, thereby compensating for subject-dependent physiological variability [S1-S3]. Depending on the underlying function structure:

$$Calibrated BP= f_{\theta}(x^{*},\Phi^{*})$$

where $Calibrated BP$ typically from validated cuff-based devices or reference-grade instruments, x*denotes personalized or recalibrated input signals, Φ∗ includes subject-specific physiological constants obtained via calibration.

In physics or physiological informed models, calibration focuses on estimating Φ∗ while keeping the structural form fθ and its associated constants θ unchanged. Common calibration strategies include: Static Parameter Calibration relies on one-time reference measurements (e.g., baseline PTT and BP) to estimate parameters Φ through directly substituting into analytical equations, assuming that these parameters remain constant over time [S4-S6]. While computationally efficient and easy to implement, it lacks adaptability to dynamic physiological changes. Personalized Model Fitting employs individual historical or longitudinal data to dynamically optimize Φ within hemodynamic equations by minimizing prediction error. Common techniques include least-squares optimization, regression-based fitting, or iterative updating within hemodynamic equations [S1, S7, S8]. For example, in the Moens-Korteweg formulation, coefficients (e.g., a, b, and γ) representing arterial wall properties can be personalized through model fitting. This approach achieves higher individualization and better long-term accuracy but increases data requirements and computational complexity. Rule-based optimization incorporates physiological priors (e.g., the relationship between heart rate, vascular compliance, and BP) to guide the adjustment of Φ without directly relying on BP labels [S9, S10]. For example, if an increase in heart rate physiologically implies an increase in SBP, model parameters can be adjusted to maintain consistency with expected cardiovascular dynamics [S3]. This method offering improved interpretability and robustness, though it may be limited by its dependency on predefined rule validity across individuals.

In ML-based models, calibration can be performed on both adjusting the subject-specific variables Φ and fine-tuning parts of the model parameters θ. Common calibration strategies include: Supervised Fine-Tuning uses a small amount of subject-specific labeled BP data to adapt pre-trained model [S11-S13]. Typically, only a subset of the θ (e.g., final layers or specific modules) is updated to better capture individual characteristics while minimizing the risk of overfitting. This method enables high personalization and improved accuracy, especially for long-term or ambulatory monitoring. However, it requires multiple cuff-based reference BP measurements, which may limit its scalability in real-world applications. Personalized Bias Correction offers a lightweight alternative by introducing a subject-dependent bias term (e.g., $\hat{BP}=f_{\theta}\left( x \right)+b_{subject}$) during inference without modifying the base model [S14-S16]. The bias is typically estimated using a few reference BP measurements and serves to correct systematic deviations in predictions. This method is computationally efficient and suitable for wearable scenarios with minimal data, though it cannot model non-linear individual-specific dynamics or long-term drift. Domain Adaptation calibrates the model by aligning feature distributions between training and target domains (e.g., different users or devices) [S17] without relying on labeled BP data. Techniques like adversarial learning feature alignment and statistical distribution matching are employed to minimize domain discrepancies. This strategy is especially valuable for scaling BP models across diverse populations or hardware platforms, though it introduces greater training complexity and requires careful regularization to maintain stability. Advanced strategies include meta-learning, which equips the model with rapid adaptation capabilities using limited data, and multimodal context-aware modeling, where demographic and behavioral factors are incorporated into the input space or latent representations. These techniques enable flexible and data-efficient personalization without compromising the model’s generalizability.

1. **Calibration Strategies Across Paradigms**

Despite methodological differences, both physics or physiology-informed and ML-based models support three calibration paradigms, each offering a distinct balance between scalability and individualization.

Population calibration adopts average physiological or model parameters Φ derived from a group of subjects and applies them uniformly across individuals. Estimations are based solely on sensor data acquired at the measurement time (or during a ‘measurement period’). While this approach ensures simplicity, efficiency, and scalability, it often fails to capture inter-individual variability, especially for subjects whose physiology deviates from population norms [S18]. For example, using fixed population-averaged PWV/PTT values [S3-S5, S8] and commonly training a single universal model across heterogeneous dataset without personalization [S16, S19-S21].

Personalized calibration targets subject-specific parameter adaptation, adjusting Φ or θ using individualized reference BP measurements [S9, S22, S23]. Personalization offers higher accuracy by explicitly modeling physiological heterogeneity but requires more data per subject, increased computational cost, and can face challenges in large-scale deployments. Representative examples include hemodynamic models fitted using initial cuff-based BP and impedance signals [S9] and [S22] fine-tune model weights or inject personal features (age, skin tone, etc.) into the input space.

Hybrid calibration combines the strengths of both paradigms by initializing from a population-trained model and applying lightweight, subject-specific adaptation [S12, S22, S24, S25]. Techniques in this category include transfer learning, adaptive parameter weighting between population and individual values [S26] or incremental self-calibration over time using unlabeled or semi-labeled data [S27].

## Note S2 Deployment factors and the algorithm–hardware selection

From the model perspective, deployability is governed by input configuration (sampling rate, window length, number of channels), model architecture (depth, width, parameters), and performance target (accuracy, latency, robustness). Consistent with the formalization in Section 3, where BP is modeled as $BP= f_{\theta}(x,\Phi)$, both the resolution and cardinality of inputs (e.g., PTT timing tied to sampling rate) and the parameter count and activation flow of models directly influence inference latency, memory consumption, and energy efficiency. Optimization strategies such as quantization, sparsity, operator fusion, causal inference, and dynamic scheduling can significantly reduce memory and compute footprints, enabling edge deployment even under tight power and latency budgets.

Hardware-side limitations are governed by on-chip SRAM/Flash capacity, compute throughput and available accelerators (MCU/NPU/DSP), along with compiler/kernel maturity and toolchain support, system-level power/energy budgets, analog front end (AFE)/ADC bandwidth, I/O and storage, and the cumulative overheads of preprocessing, loading, inference, and persistent state.

Based on **Table 2** and **Table S2**, low-complexity models including physiological informed models, traditional ML models and lightweight neural networks can be executed entirely on ultra-low-power microcontrollers (MCUs) or compact SoCs embedded in wearables, typically ~10–200 KB Flash and ~1–5 KB SRAM, with <5 ms inference at 64–160 MHz and negligible incremental energy. Mid-complexity models such as compact deep networks, benefit from the higher compute and memory available in edge platforms like smartphones. With int8 quantization, models of ~20–500 k parameters sustain streaming inference, on M55 + micro-NPU (e.g., U55/U65-class) latency is ~1–5 ms per window, enabling real-time and beat-synchronous pipelines. High-complexity models including transformers and foundation models, demand extensive compute and memory resources, typically require deployment in edge–cloud or cloud-only systems with access to dedicated AI accelerators.

**On-device Computing** refers to executing the entire signal processing and inference pipeline locally on the wearable device using resource-limited MCUs or ultra-low-power system-on-chips (SoCs). Representative platforms include the Nordic nRF52840, ESP32-S3, and STM32N6, paired with energy-efficient AFEs. Low-complexity models are commonly employed, with emerging approaches leveraging TinyML frameworks (e.g., TFLite Micro). This paradigm offers strong advantages in privacy, latency, and energy efficiency, well-suited for deployment in low-infrastructure or LMIC contexts [S28, S29].

**Device-Edge Collaboration** partitions preprocessing to the wearable devices and offloads complex inference to nearby edge nodes (e.g. smartphone, gateways). This approach supports multimodal data analysis and real-time feedback, reducing cloud dependency while leveraging more capable SoCs with integrated NPUs or DSPs (e.g., Canaan K510, Rockchip RK3588) for intermediate AI workloads. It strikes a balance between performance and efficiency but requires stable local connectivity [S30].

**Device-Edge-Cloud Collaboration** enables local preprocessing and lightweight inference while offloading complex computations to the cloud, supporting advanced inference (e.g., transformers, federated learning). Cloud-level deployment benefits from high-performance accelerators such as TPUs, FPGAs, and custom ASICs that support scalable training and cross-device personalization. While it supports powerful analytics and global optimization, it introduces complexity, latency, and strong reliance on connectivity [S31].

## Note S3 Hardware System Design

To implement the reliable, real-time BP estimation on wearable devices, it requires optimized hardware design across the analog front-end (AFE) and digital processing. The AFE is responsible for signal acquisition and analog-to-digital conversion, while the computing subsystem executes AI inference.

**1) AFE Design for Ultra-Low-Power Biosignal Acquisition**

Given the microampere-level signal amplitude and stringent power budgets, AFE design becomes a critical bottleneck for system scalability and real-world deployment. Traditional AFE is facing the challenging for its high-power consumption and limited DR. Commercial AFEs such as the **TI AFE4900** and **ADI MAX86176**, which typically operate in the **200–300 µW** range and lack dynamic gain control. Recent efforts have introduced light-to-digital converter (LDC) architectures to directly integrate photocurrent and reduce analog complexity. A representative design is the SAR-LDC, which uses a current-integration SAR quantizer to achieve sub-10 µW power consumption and a dynamic range of up to 148.9 dB [S32]. The design allows for deactivation of the integrator during conversion, significantly reducing phase power and noise. These developments underscore the critical role of **AFE–digital co-design** in enabling scalable, ultra-low-power signal acquisition, forming the foundation for continuous and reliable wearable BP monitoring systems.

**2) Digital Computing Platforms**

Following signal acquisition, efficient processing of BP estimation models requires carefully selected computing hardware that balances power, latency, and model complexity. Depending on application scenarios and resource budgets, digital processing platforms can be categorized into three levels (Table 2):

Wearable device (MCUs): Ultra-low-power MCUs like Nordic nRF52840 and ESP32-S3 integrate CPU, memory, and peripheral interfaces into a compact architecture. They provide high-bandwidth transmission (e.g., 2.4 GHz and 5 GHz), enabling edge-cloud collaboration for vital signal processing. Advanced MCUs (e.g., Exynos W1000, STM32N6 with on-chip NPUs) operate at higher clock speeds and support lightweight AI frameworks for on-device health analytics.

Edge device (SoCs): System-on-Chip (SoC) found in smartphones or edge hubs integrated CPUs, Graphics Processing Unit (GPUs), Digital Signal Processor (DSPs), memory, wireless modules, and AI accelerators on a single chip. Advanced SoCs with integrated NPUs deliver several to dozens of TOPS and support higher DDR RAM capacities (up to several GB), outperforming wearable processing units. SoCs like the Canaan K510 also accommodate diverse AI frameworks (e.g., TensorFlow, ONNX) and data types (e.g., BF16), making them ideal for deploying complex BP estimation models with multimodal input.

Specialized AI Accelerators: Emerging dedicated AI hardware accelerators such as GPUs, Field Programmable Gate Array (FPGAs) and Application-Specific Integrated Circuit (ASICs) typically designed with specialized instruction sets and architectures such as tensor processing cores (TPUs), neural processing units (NPUs), vision processing units (VPUs) and low-power designs for AI-specific foperations. These accelerators offer exceptional computational performance—ranging from several to dozens of TFLOPS per module—alongside larger RAM, making them well-suited for intensive tasks like personalized model training. Though not suited for wearables, they are ideal for edge-cloud scenarios where real-time responsiveness and scalability are critical.

Table S1 Comparative analysis of the advantages and trade-offs between the physics or physiological BP models

| **Model** | **Advantages** | **Trade-Offs** | **Best Use Case** |
| --- | --- | --- | --- |
| **Windkessel** | Simple, computationally efficient, suitable for real-time monitoring. | Oversimplifies arterial dynamics, less accurate for complex systems. | Wearables, basic clinical monitoring. |
| **Arterial Wall Mechanics** | High accuracy for biomechanical analysis, captures vessel-specific properties. | Requires advanced imaging, not scalable for low-cost applications. | Research, diagnostic vascular studies. |
| **Pulse Wave Propagation** | Non-invasive, adaptable to wearables, good for continuous monitoring. | Sensitive to noise and anatomical variations, moderate accuracy. | Wearables, non-invasive monitoring. |
| **PINNs** | Handles complex dynamics, integrates data and physics for interpretability. | High computational cost, limited real-time use, complex implementation. | Advanced research, precision medicine. |

Table S2 The detailed specs on representative computing chips

|  | Device | Architecture | Compute power/Clock frequency | Power consumption (W) | Power efficiency (TOPS/W) | Memory |
| --- | --- | --- | --- | --- | --- | --- |
| Wearable devices | Nordic nRF52840 | CPU | 64MHz | 0.0165 | - | 1MB |
|  | ESP32 S3 | CPU | 240MHz | - | - | 512KB |
|  | NXP MIMXRT595SFFOC | CPU+GPU+DSP | 275MHz | 0.05 |  | 5MB |
|  | Ambiq Apollo510 | CPU+GPU | 250MHz | 0.0014 | - | 2MB |
|  | Exynos W1000 | CPU+GPU | 1.6GHz | - | - | 16GB |
|  | STM32N6 | CPU+NPU | 0.6/1Ghz | <1 | 0.5 | 4.2MB |
|  | Raspberry Pi 4 | CPU+GPU | 1.5GHz | 3.8-4 | - | 256KB |
| Edge devices | Cannan K510 | CPU+GPU+DSP+KPU | 3 TOPS | 2 | 1.5 | 512MB |
|  | Rockchip RK3588 | CPU+GPU+DSP+NPU | 6 TOPS | 5-10 | 0.6-1.2 | 8GB |
|  | MediaTek Dimensity 9300 | CPU+GPU+DSP+APU | ~48 TOPS | 2-10 | 5 | DDR RAM |
|  | Snapdragon 8 gen3 | CPU+GPU+DSP+NPU | ~73 TOPS | 2-10 | 2.5 | 8GB |
| AI accelerators | Jetson Nano | CPU+GPU | 0.5 TOPS | 5-10 | 0.05-0.1 | 4GB |
|  | Movidius Myriad X | VPU | 1 TOPS | 1 | 1 | 4GB |
|  | Google Edge TPU | TPU | 4 TOPS | 2 | 2 | 1GB |
|  | Xilinx Zynq ZU9 | CPU+FPGA | 4.1 TOPS | 15 | 0.273 | 512KB |
|  | Ascend 310 | CPU+GPU+NPU | 16 TOPS | 8 | 0.5 | 8GB |
|  | Jetson Xavier | CPU+GPU+DSP | 21 TOPS | 10-15 | 1.4-2.1 | 8/16GB |
|  | Halio 8 | NPU | 26 TOPS | 2.5 | 10.4 | On chip |

Table S3 Trade-offs between BP reporting frequency, clinical applications, and deployment constraints in resource-limited settings

| **Application Scenario** | **Target Population** | **Typical Frequency** | **Suggested Deployment** |
| --- | --- | --- | --- |
| High-Demand  (Acute Care) | Intraoperative, ICU, drug testing, elderly/hypertensive at night | BtB/every 1–5 min | Always-on inference, synchronized multi-sensor input, edge/cloud hybrid |
| Moderate-Demand (Monitoring & Risk) | At-risk populations, post-discharge, ambulatory care | Every 30–60 min | Duty-cycled sensing, lightweight models, on-device preferred |
| Low-Demand (Wellness & Lifestyle) | Healthy users, fitness tracking, population-scale screening | Daily/weekly/monthly | Statistical smoothing, daily averages, ultra-low-power mode |

# Supplementary References

1. R. Mukkamala, J.-O. Hahn, O.T. Inan, L.K. Mestha, C.-S. Kim et al., Toward ubiquitous blood pressure monitoring *via* pulse transit time: theory and practice. IEEE Trans. Biomed. Eng. **62**(8), 1879–1901 (2015). <https://doi.org/10.1109/TBME.2015.2441951>
2. D. Barvik, M. Cerny, M. Penhaker, N. Noury, Noninvasive continuous blood pressure estimation from pulse transit time: a review of the calibration models. IEEE Rev. Biomed. Eng. **15**, 138–151 (2022). <https://doi.org/10.1109/RBME.2021.3109643>
3. W. Chen, T. Kobayashi, S. Ichikawa, Y. Takeuchi, T. Togawa, Continuous estimation of systolic blood pressure using the pulse arrival time and intermittent calibration. Med. Biol. Eng. Comput. **38**(5), 569–574 (2000). <https://doi.org/10.1007/BF02345755>
4. C.C.Y. Poon, Y.T. Zhang, Cuff-less and noninvasive measurements of arterial blood pressure by pulse transit time. 2005 IEEE Engineering in Medicine and Biology 27th Annual Conference., 5877–5880. IEEE (2006).
5. X.-R. Ding, Y.-T. Zhang, J. Liu, W.-X. Dai, H.K. Tsang, Continuous cuffless blood pressure estimation using pulse transit time and photoplethysmogram intensity ratio. IEEE Trans. Biomed. Eng. **63**(5), 964–972 (2016). <https://doi.org/10.1109/TBME.2015.2480679>
6. T.H. Huynh, R. Jafari, W.-Y. Chung, Noninvasive cuffless blood pressure estimation using pulse transit time and impedance plethysmography. IEEE Trans. Biomed. Eng. **66**(4), 967–976 (2019). <https://doi.org/10.1109/TBME.2018.2865751>
7. S. Qiu, Y.-T. Zhang, S.-K. Lau, N. Zhao, Scenario adaptive cuffless blood pressure estimation by integrating cardiovascular coupling effects. IEEE J. Biomed. Health Inform. **27**(3), 1375–1385 (2023). <https://doi.org/10.1109/JBHI.2022.3227235>
8. J. Liu, B.P. Yan, Y.-T. Zhang, X.-R. Ding, P. Su et al., Multi-wavelength photoplethysmography enabling continuous blood pressure measurement with compact wearable electronics. IEEE Trans. Biomed. Eng. **66**(6), 1514–1525 (2019). <https://doi.org/10.1109/TBME.2018.2874957>
9. K. Sel, A. Mohammadi, R.I. Pettigrew, R. Jafari, Physics-informed neural networks for modeling physiological time series: a case study with continuous blood pressure. Res. Sq., rs.3.rs–rs.2423200 (2023). <https://doi.org/10.21203/rs.3.rs-2423200/v1>
10. R. Wang, M. Qi, Y. Shao, A. Zhou, H. Ma, PITN: physics-informed temporal networks for cuffless blood pressure estimation. arXiv: 2408.08488 (2024). <https://doi.org/10.48550/arXiv.2408.08488>
11. Y. Zhang, X. Ren, X. Liang, X. Ye, C. Zhou, A refined blood pressure estimation model based on single channel photoplethysmography. IEEE J. Biomed. Health Inform. **26**(12), 5907–5917 (2022). <https://doi.org/10.1109/JBHI.2022.3206477>
12. J. Leitner, P.-H. Chiang, S. Dey, Personalized blood pressure estimation using photoplethysmography: a transfer learning approach. IEEE J. Biomed. Health Inform. **26**(1), 218–228 (2022). <https://doi.org/10.1109/JBHI.2021.3085526>
13. W. Wang, P. Mohseni, K.L. Kilgore, L. Najafizadeh, Cuff-less blood pressure estimation from photoplethysmography *via* visibility graph and transfer learning. IEEE J. Biomed. Health Inform. **26**(5), 2075–2085 (2022). <https://doi.org/10.1109/JBHI.2021.3128383>
14. S. Haddad, A. Boukhayma, A. Caizzone, Continuous PPG-based blood pressure monitoring using multi-linear regression. IEEE J. Biomed. Health Inform. **26**(5), 2096–2105 (2022). <https://doi.org/10.1109/JBHI.2021.3128229>
15. O. Schlesinger, N. Vigderhouse, D. Eytan, Y. Moshe, Blood pressure estimation from PPG signals using convolutional neural networks and Siamese network. ICASSP 2020 - 2020 IEEE International Conference on Acoustics, Speech and Signal Processing (ICASSP). May 4-8, 2020. Barcelona, Spain. IEEE, (2020).: 1135-1139. <https://doi.org/10.1109/icassp40776.2020.9053446>
16. M. Simjanoska, M. Gjoreski, M. Gams, A. Madevska Bogdanova, Non-invasive blood pressure estimation from ECG using machine learning techniques. Sensors **18**(4), 1160 (2018). <https://doi.org/10.3390/s18041160>
17. A. Cisnal, Y. Li, B. Fuchs, M. Ejtehadi, R. Riener et al., Robust feature selection for BP estimation in multiple populations: towards cuffless ambulatory BP monitoring. IEEE J. Biomed. Health Inform. **28**(10), 5768–5779 (2024). <https://doi.org/10.1109/JBHI.2024.3411693>
18. A. Avolio, J. Cox, K. Louka, F. Shirbani, I. Tan et al., Challenges presented by cuffless measurement of blood pressure if adopted for diagnosis and treatment of hypertension. Pulse **10**(1–4), 34–45 (2022). <https://doi.org/10.1159/000522660>
19. M.H. Chowdhury, M.N.I. Shuzan, M.E.H. Chowdhury, Z.B. Mahbub, M.M. Uddin et al., Estimating blood pressure from the photoplethysmogram signal and demographic features using machine learning techniques. Sensors **20**(11), 3127 (2020). <https://doi.org/10.3390/s20113127>
20. T. Xiang, Y. Zhang, Y. Zhang, A novel multimodal physiological model for the noninvasive and continuous measurements of arterial blood pressure. 2024 46th Annual International Conference of the IEEE Engineering in Medicine and Biology Society (EMBC)., 1–6. IEEE (2024).
21. M.S. Roy, R. Gupta, K. Das Sharma, BePCon: a photoplethysmography-based quality-aware continuous beat-to-beat blood pressure measurement technique using deep learning. IEEE Trans. Instrum. Meas. **71**, 2519709 (2022). <https://doi.org/10.1109/TIM.2022.3212750>
22. Y. Zhang, C. Zhou, X. Ren, Q. Wang, H. Wang et al., Personalized continuous blood pressure tracking through single channel PPG in wearable scenarios. IEEE J. Biomed. Health Inform. **29**(6), 4109–4120 (2025). <https://doi.org/10.1109/JBHI.2025.3535788>
23. C. Yao, T. Sun, S. Huang, M. He, B. Liang et al., Personalized machine learning-coupled nanopillar triboelectric pulse sensor for cuffless blood pressure continuous monitoring. ACS Nano **17**(23), 24242–24258 (2023). <https://doi.org/10.1021/acsnano.3c09766>
24. Y. Zhang, X. Ren, X. Liang, X. Ye, C. Zhou, A refined blood pressure estimation model based on single channel photoplethysmography. IEEE J. Biomed. Health Inform. **26**(12), 5907–5917 (2022). <https://doi.org/10.1109/JBHI.2022.3206477>
25. J. Chen, X. Zhou, L. Feng, B.W. Ling, L. Han et al., rU-net, multi-scale feature fusion and transfer learning: unlocking the potential of cuffless blood pressure monitoring with PPG and ECG. IEEE J. Biomed. Health Inform. **29**(1), 166–176 (2025). <https://doi.org/10.1109/JBHI.2024.3483301>
26. Z.-D. Liu, Y. Li, Y.-T. Zhang, J. Zeng, Z.-X. Chen et al., HGCTNet: handcrafted feature-guided CNN and transformer network for wearable cuffless blood pressure measurement. IEEE J. Biomed. Health Inform. **28**(7), 3882–3894 (2024). <https://doi.org/10.1109/JBHI.2024.3395445>
27. H. Chen, Z. Zeng, Y. Zhang, L. Lyu, R.H.M. Chan et al., Multi-wavelength photoplethysmographic assessment of fingertip vasculature for auto-calibrated cuffless blood pressure monitoring. IEEE J. Biomed. Health Inform. DOI:10.1109/JBHI.2025.3587042 (2025). <https://doi.org/10.1109/JBHI.2025.3587042>
28. O. Durmaz Incel, S.Ö. Bursa, On-device deep learning for mobile and wearable sensing applications: a review. IEEE Sens. J. **23**(6), 5501–5512 (2023). <https://doi.org/10.1109/JSEN.2023.3240854>
29. N.N. Alajlan, D.M. Ibrahim, TinyML: enabling of inference deep learning models on ultra-low-power IoT edge devices for AI applications. Micromachines **13**(6), 851 (2022). <https://doi.org/10.3390/mi13060851>
30. X. Wang, Y. Han, V.C.M. Leung, D. Niyato, X. Yan et al., Convergence of edge computing and deep learning: a comprehensive survey. IEEE Commun. Surv. Tutor. **22**(2), 869–904 (2020). <https://doi.org/10.1109/COMST.2020.2970550>
31. W. Goossens, D. Mustefa, D. Scholle, H. Fotouhi, J. Denil, Evaluating edge computing and compression for remote cuff-less blood pressure monitoring. J. Sens. Actuator Netw. **12**(1), 2 (2023). <https://doi.org/10.3390/jsan12010002>
32. Y.-H. Gao, J. Li, C.-H. Fan, K.N. Leung, Y.-T. Zhang et al., A 9.84-μW 148.9-dB total DR light-to-digital converter with current-integration SAR quantizer for multi-wavelength PPG applications. IEEE Trans. Circuits Syst. I Regul. Pap. **72**(11), 6886–6899 (2025). <https://doi.org/10.1109/TCSI.2025.3564384>
